# Human adipose-derived mesenchymal stem cell-conditioned medium ameliorates polyneuropathy and foot ulceration in diabetic BKS db/db mice

**DOI:** 10.1186/s13287-020-01680-0

**Published:** 2020-05-01

**Authors:** Cristian De Gregorio, David Contador, Diego Díaz, Constanza Cárcamo, Daniela Santapau, Lorena Lobos-Gonzalez, Cristian Acosta, Mario Campero, Daniel Carpio, Caterina Gabriele, Marco Gaspari, Victor Aliaga-Tobar, Vinicius Maracaja-Coutinho, Marcelo Ezquer, Fernando Ezquer

**Affiliations:** 1 Center for Regenerative Medicine, School of Medicine Clínica Alemana-Universidad del Desarrollo, Av. Las Condes 12438, Lo Barnechea, Santiago, Chile; 2 Institute of Histology and Embryology of Mendoza (IHEM-CONICET), School of Medicine, Universidad Nacional de Cuyo, Mendoza, Argentina; 3 Department of Neurology & Neurosurgery, Hospital José Joaquín Aguirre, Universidad de Chile, Santiago, Chile; 4 Institute of Anatomy, Histology and Pathology, Universidad Austral de Chile, Valdivia, Chile; 5 Research Center for Advanced Biochemistry and Molecular Biology, Department of Experimental and Clinical Medicine, University of Catanzaro, Catanzaro, Italy; 6 Advanced Center for Chronic Diseases-ACCDiS, Faculty of Chemical and Pharmaceutical Sciences, Universidad de Chile, Santiago, Chile

**Keywords:** Diabetic polyneuropathy, Diabetic foot ulcer, Mesenchymal stem cells, Conditioned medium, Deferoxamine

## Abstract

**Background:**

Diabetic polyneuropathy (DPN) is the most common and early developing complication of diabetes mellitus, and the key contributor for foot ulcers development, with no specific therapies available.

Different studies have shown that mesenchymal stem cell (MSC) administration is able to ameliorate DPN; however, limited cell survival and safety reasons hinder its transfer from bench to bedside. MSCs secrete a broad range of antioxidant, neuroprotective, angiogenic, and immunomodulatory factors (known as conditioned medium), which are all decreased in the peripheral nerves of diabetic patients. Furthermore, the abundance of these factors can be boosted in vitro by incubating MSCs with a preconditioning stimulus, enhancing their therapeutic efficacy. We hypothesize that systemic administration of conditioned medium derived from preconditioned MSCs could reverse DPN and prevent foot ulcer formation in a mouse model of type II diabetes mellitus.

**Methods:**

Diabetic BKS *db/db* mice were treated with systemic administration of conditioned medium derived from preconditioned human MSCs; conditioned medium derived from non-preconditioned MSCs or vehicle after behavioral signs of DPN was already present. Conditioned medium or vehicle administration was repeated every 2 weeks for a total of four administrations, and several functional and structural parameters characteristic of DPN were evaluated. Finally, a wound was made in the dorsal surface of both feet, and the kinetics of wound closure, re-epithelialization, angiogenesis, and cell proliferation were evaluated.

**Results:**

Our molecular, electrophysiological, and histological analysis demonstrated that the administration of conditioned medium derived from non-preconditioned MSCs or from preconditioned MSCs to diabetic BKS *db/db* mice strongly reverts the established DPN, improving thermal and mechanical sensitivity, restoring intraepidermal nerve fiber density, reducing neuron and Schwann cell apoptosis, improving angiogenesis, and reducing chronic inflammation of peripheral nerves. Furthermore, DPN reversion induced by conditioned medium administration enhances the wound healing process by accelerating wound closure, improving the re-epithelialization of the injured skin and increasing blood vessels in the wound bed in a skin injury model that mimics a foot ulcer.

**Conclusions:**

Studies conducted indicate that MSC-conditioned medium administration could be a novel cell-free therapeutic approach to reverse the initial stages of DPN, avoiding the risk of lower limb amputation triggered by foot ulcer formation and accelerating the wound healing process in case it occurs.

## Background

Diabetic polyneuropathy (DPN) is the most common and early developing complication of diabetes mellitus (DM), affecting up to 60% of type I and type II DM patients [1]. It is characterized by progressive distal-to-proximal degeneration of peripheral nerves, resulting in sensory loss, chronic pain, and recurrent ulcerations [2, 3]. These ulcerations do not proceed through the normal wound healing process and often develop gangrenous infections, leading to the amputation of the affected limb [2]. Thus, DPN is responsible for 20% of the hospitalizations of DM patients and is the second leading cause of amputation worldwide [4].

Despite the continuing and increasing incidence of DPN, nowadays, there is no specific treatment able to stop DPN progression and prevent the formation of foot ulcers (FU). Furthermore, the palliative drugs commonly used, such as antiepileptics, antidepressants, and opioids, are symptomatic treatments with untoward side effects; thus, they are not recommended as long-term treatments [5]. The main difficulty in generating a comprehensive treatment for DPN is due to its highly complex and multifactorial etiology [6]. Strong evidence shows that exacerbated glucose flux leads to the over-activation of different pathways for its metabolism. The common factor of these pathways is the production of high levels of reactive oxygen species (ROS) in the mitochondria, turning oxidative stress into a central player in the onset and progression of DPN [7]. Increased ROS levels induce (i) apoptosis of neurons and Schwann cells in dorsal root ganglia (DRG) and peripheral nerves, with a concomitant reduction in the local levels of neuroprotective factors; (ii) reduction of the local blood flow to nerve fibers and a marked decline in the angiogenic process; and (iii) a chronic pro-inflammatory process [6, 8]. Therefore, the reduced expression of neuroprotective, angiogenic, and anti-inflammatory factors in peripheral nerves plays a crucial role in the development and progression of DPN [6, 8]. In FU in diabetic patients, the detailed mechanisms of impaired wound healing are not completely understood. However, it is clear that the degeneration of nerve fibers and the reduced blood flow greatly contribute to this process [2].

Based on its various causative pathways, a number of experimental strategies to treat DPN have focused on the systemic administration of antioxidant [9], anti-inflammatory [10], neuroprotective [11], and angiogenic factors [12]. However, while most of these strategies have shown promising results in preclinical animal studies, the transfer of these procedures to clinical practice has been unsuccessful [8, 13], which is mainly related to the fact that the pathogenesis of DPN is multifactorial. Therefore, a single molecule-based therapy (mono-target therapy) is unlikely to be effective.

Stem cell-based therapies represent promising therapeutic approaches for the treatment of complex diseases like DPN, since unlike conventional pharmacological treatments, stem cells act through multiple mechanisms (multi-target therapy) [14]. Among the different types of adult stem cells, adipose tissue-derived mesenchymal stem cells (AD-MSCs) have several advantages that make them attractive candidates for the treatment of DPN, since they (i) can be easily isolated from the waste material of cosmetic liposuction and highly expanded in vitro, (ii) secrete several antioxidant molecules [15], and (iii) produce high levels of trophic factors including neuroprotective, angiogenic, and anti-inflammatory factors [16, 17].

In this sense, it has been reported that the intramuscular injection of MSCs into animal models of DPN induced the recovery of motor and sensory nerve conduction velocities, the recovery of the normal ultrastructure of peripheral nerves, the induction of an increased blood flow to damaged nerves, and a reduction in the hypoalgesia to thermal and mechanical stimulation [18–21]. In these reports, the therapeutic effects were correlated with an increase in the levels of several neuroprotective, angiogenic, and anti-inflammatory factors in the nerves, with no differentiation of MSCs into neurons or Schwann cells [18–20]. However, the clinical use of MSCs is still reduced, due to several safety and technical limitations associated with the administration of living cells [22–24].

In the case of FU, it has also been reported that the administration of MSCs directly onto the wounds improved wound closure by promoting skin re-epithelialization and improving the angiogenic process [25, 26]. However, the primary limitation of this therapeutic strategy is that since DPN persists, FU are constantly occurring in diabetic patients. Therefore, this complex procedure needs to be repeated whenever the patient has a new ulcer.

The main therapeutic mechanism associated with MSC administration is the paracrine secretion of a broad spectrum of biologically active factors and extracellular vesicles, generally referred to as MSC-conditioned medium [27]. Furthermore, it has been reported that the abundance of these molecules can be regulated in vitro by incubating the cells with a preconditioning stimulus [28], enriching the composition of the conditioned medium with molecules relevant for the treatment of a specific pathology. Thus, MSC-derived conditioned medium could be used as a biodrug, replacing the need to administer living cells. Furthermore, this treatment can be repeated over time since it does not generate immunological reactions [29].

Recently, we reported that the preconditioning of AD-MSC by in vitro incubation with the hypoxic mimetic agent deferoxamine (DFX) induced a significant increase in the secretion of potent antioxidant, neuroprotective, angiogenic, and anti-inflammatory factors compared to non-preconditioned cells [30]. Additionally, conditioned medium obtained from DFX preconditioned cells showed greater neuroprotective effects compared with the conditioned medium obtained from non-preconditioned MSCs, when evaluated in an in vitro model of DPN [30].

In the present study, we evaluated whether the systemic administration of conditioned medium derived from DFX-preconditioned AD-MSCs or from non-preconditioned AD-MSCs was able to reverse several functional and structural alterations characteristic of DPN and to prevent the formation of FU, in BKS db/db mice, one of the most robust animal models of DPN [31–33]. We demonstrated that systemic administrations of conditioned medium derived from non-preconditioned MSCs or from DFX-preconditioned MSCs strongly revert the established DPN, improving thermal and mechanical sensitivity, restoring normal skin innervation, reducing sensory neuron and Schwann cell apoptosis, improving angiogenesis, and reducing chronic inflammation. Furthermore, DPN reversion induced by conditioned medium administration enhances the wound healing process; thus, this therapy could also be effective in preventing FU formation in diabetic patients.

## Methods

### Animals

Transgenic mice (BKS.Cg-m+/+Lepr^db^/J) that spontaneously develop T2DM were purchased from Jackson Laboratories (Bar Harbor, USA). Female diabetic (*db/db*) and non-diabetic (*db/+*) mice were housed at constant temperature and humidity, with a 12-h light/dark cycle and unrestricted access to standard chow and water. All animal protocols were approved by the Ethics Committee of Universidad del Desarrollo.

### Isolation, expansion, and characterization of AD-MSCs

Human AD-MSCs were isolated from fresh subcutaneous adipose tissue samples (abdominal region) obtained from liposuction aspirates of four healthy female donors undergoing cosmetic liposuction at Clínica Alemana, Chile, as previously described [30]. Written informed consent was obtained for all samples and protocols used were approved by the Ethics Committee of Universidad del Desarrollo. After two subcultures, adherent cells were characterized according to their adipogenic and osteogenic differentiation potential, by the presence of putative MSC markers (CD29, CD13, CD105, CD73, and CD90) and the absence of markers characteristic of other cell lineages (CD235a, CD31, and CD45) as previously described [30].

### Preconditioning of AD-MSC with DFX and conditioned medium purification

AD-MSCs (passage 3) were plated at a density of 7000 cells/cm^2^ and cultured in minimum essential medium (α-MEM) (Gibco, New Zealand) supplemented with 10% fetal bovine serum (FBS) (Gibco, New Zeeland) and 0.16 mg/ml gentamicin (Sanderson Laboratory, Chile) at 37°C in a 5% CO_2_ atmosphere. When cells reached 70% confluence, plates were rinsed three times with PBS and cells were incubated for 48 h in α-MEM without FBS supplemented with 400 μM DFX (Sigma-Aldrich, USA) (preconditioned AD-MSCs) or with saline (vehicle) as non-preconditioned AD-MSCs. After the incubation with DFX or saline, the MSC-conditioned media were obtained by harvesting the culture media. The media were centrifuged at 400*g* for 10 min to remove whole cells, and the supernatant was centrifuged again at 5000 g for 20 min to remove cell debris as previously described [30]. Finally, conditioned media were filtered in 0.22-μm filters and concentrated 10 times (v/v) using 3 kDa cutoff filters (Millipore, USA). To completely eliminate DFX from the conditioned medium, the concentrates were washed twice with 15 ml of PBS and re-concentrated using the same filters.

### Systemic administration of MSC-derived conditioned medium

At 18 weeks of age, diabetic (*db/db*) mice were randomly divided into three groups. The first group of mice was treated with intravenous administration of 50 μl of conditioned medium derived from 1 × 10^6^ DFX-preconditioned AD-MSCs. The second group was treated with intravenous administration of 50 μl of conditioned medium derived from 1 × 10^6^ non-preconditioned AD-MSCs, while the third group received intravenous administration of 50 μl of vehicle. Conditioned medium or vehicle administration was repeated every 2 weeks for a total of four administrations. An additional group of normal non-diabetic (*db/+*) mice treated with vehicle was used as a healthy control (Supplementary Figure 1).

### Measurement of blood glucose

Non-fasting blood glucose levels were measured using a glucometer (Accu-Chek Performa System; Roche, Switzerland). Blood samples were obtained from the tail of alert animals every 2 weeks (from week 14 to week 26).

### Determination of thermal sensitivity

At 14, 18, 22, and 26 weeks of age, mice were placed in an acrylic box provided with an infrared light (Ugo Basile Plantar Test, Italy) 20 min before measuring the withdrawal latency. Then, the infrared light was placed beneath the mid-plantar surface of hind paws, and the withdrawal responses were automatically recorded. The cutoff latency was set at 15 s to avoid hind paw damage. The stimulation was repeated three times with an interval of 5 min between stimuli, during two consecutive days. Eight animals per experimental group were evaluated. Data were expressed as the mean withdrawal latency registered each day [33].

### Determination of mechanical sensitivity

At 14, 18, 22, and 26 weeks of age, mice were placed in an acrylic box with a mesh floor that allowed free access to the plantar surface of the paw 20 min before evaluation. Then, the mid-plantar surface of hind paws was stimulated with an electronic Von Frey filament (Ugo Basile Electronic Von Frey, Italy) with increasing force, and the responses were automatically recorded. The stimulation was repeated three times with an interval of 5 min between stimuli, during two consecutive days. Eight animals per experimental group were evaluated. Data were expressed as the mean withdrawal force registered each day [33].

### Quantification of nerve conduction velocity

Motor nerve conduction was determined bilaterally in the sciatic nerves. For this, animals were anesthetized by sevoflurane vapors and placed on a hot plate to maintain a constant temperature of 33 °C. For determination of motor conduction velocity, the sciatic nerve was stimulated by a supramaximal pulse of 50 μs duration with an electromyograph (Medelec, UK) with surface electrodes placed into the sciatic notch and ankle. The distance between the active and referential electrode was kept constant at 6 mm. Signals were amplified with a bandpass 2 Hz-5KHz analog filter. Signals were analyzed by Medelec software as previously described [33].

### Quantification of intraepidermal nerve fiber density

At 26 weeks of age, mice were euthanized by a ketamine/xylazine overdose. The footpads were dissected from the plantar surface of the hind paws and fixed in 4% paraformaldehyde (Merck, USA) for 15 min. The pad samples were immersed in 20% sucrose for 72 h and embedded in Tissue-Tek O.C.T. (Sakura Finetek, USA). The pads were cryosectioned at 15 μm, blocked with 5% fish gelatin, and stained against the axonal protein PGP9.5 (1:150, ab1761 Millipore, USA) as previously reported [33]. Nuclei were counterstained with DAPI (Applichem, Spain). A stack of eight images per footpad was obtained in a Fluoview FV10i confocal microscope (Olympus, Japan). Samples from six animals per experimental group were evaluated. Data were expressed as number of fibers crossing from dermis to epidermis per linear mm of skin [33].

### Morphometric analysis of sciatic nerve

At 26 weeks of age, sciatic nerves were dissected and fixed in 2.5% glutaraldehyde (Polisciences Inc., USA). Then, samples were post-fixed in 1% osmium tetroxide and embedded in epoxy resin (Sigma-Aldrich). For nerve fiber analysis, semithin (0.5 μm) transverse sections were stained with toluidine blue (Sigma-Aldrich), and the area and density of individual fibers were quantified using ImageJ software (NIH, USA). For ultrastructural analysis of the fibers, ultrathin sections were stained with 5% uranyl acetate and 3% lead citrate (Sigma-Aldrich) and examined with a Phillips Morgani electron microscope. G-ratio was analyzed as previously described [34], evaluating small caliber fibers (< 6 μm diameter) separately from large fibers (≥ 6 μm diameter). Mitochondrial area and density were quantified from electron microscopy images from sciatic nerves, using ImageJ software. Samples from six animals per experimental group were evaluated.

### Primary cultures of dorsal root ganglia (DRG) and Sholl analysis

DRGs from L3 and L4 were dissected from 26-week-old animals and enzymatically digested by incubation for 25 min at 37 °C with 0.6% trypsin (Gibco) and then for 25 min at 37 °C with 0.5% collagenase type I (Gibco). The enzymatic activity was halted by adding 1 ml of fetal bovine serum (FBS), and tissue suspension was centrifuged at 400*g* for 5 min. Then, the pellet was resuspended in DMEM/F12 (Gibco) with 10% FBS and mechanically dissociated. Five thousand cells were plated on coverslips coated with 0.05% poly-D-lysine (Sigma-Aldrich). Neurons were allowed to attach to the substratum for 4 h before changing medium to DMEM/F12 containing N2 supplement (Gibco). After 40 h in culture, cells were fixed with 4% paraformaldehyde containing 4% sucrose during 20 min, neurons were immunostained with β3-tubuline antibody (TU-20, Santa Cruz Biotechnology; secondary antibody: anti mouse AlexaFluor-488, Cell Signaling, USA), and nuclei were counterstained with DAPI. Samples were analyzed by confocal microscopy.

Sholl analysis was carried out as previously described [35]. Briefly, confocal images were transformed to 8-bit binary images, and the estimated soma center was marked. Images were analyzed with the Sholl analysis tool using ImageJ software. The distance/neurite number profile, the maximum radius reached by each neuron, and the sum of intersections for each neuron was evaluated. As positive control for neuritogenesis, samples were incubated with 10 ng/ml NGF (Alamone Labs, Israel). To avoid the quantification of neurite growth in dying neurons, we only analyzed neurons extending at least one neurite ≥ 20 μm. Samples from four animals per experimental group were evaluated.

### Determination of apoptosis in DRG and sciatic nerves

At 26 weeks of age, DRG (L3) and sciatic nerves were extracted, fixed in 4% paraformaldehyde and embedded in paraffin. Apoptotic cells were identified in 4-μm thick sections using the DeadEnd™ Fluorometric TUNEL system (Promega, USA). Nuclei were counterstained with DAPI. TUNEL-positive nuclei were quantified using ImageJ software. Samples from six animals per experimental group were evaluated. Data were expressed as the percentage of apoptotic cells [33].

### Quantification of microvasculature and T cell and macrophage infiltration in the sciatic nerve

At 26 weeks of age, sciatic nerves and gastrocnemius muscle were removed and fixed in 4% paraformaldehyde for 24 h. For microvasculature analysis, 10 μm longitudinal cryosections of sciatic nerves were obtained. Samples were permeabilized with 1 mg/ml digitonin (Calbiochem, USA) in phosphate buffer and incubated with BS1-Lectin-Alexa-647 (1:100; Life Technologies, USA) overnight. Nuclei were counterstained with DAPI. The number of BS1-Lectin-Alexa-647-positive blood vessels were quantified by confocal microscopy and normalized related to nerve area. For determination of capillary to muscle fiber ratio, muscle samples were cut into 5-μm sections and stained with hematoxylin and eosin. The number of capillaries and muscle fibers were counted in 10 fields from each section, and the capillary to muscle fiber ratio was calculated. Samples from six animals per experimental group were evaluated.

For T cell and macrophage infiltration analysis, 10-μm longitudinal nerve cryosections were blocked for 1 h in 5% FBS, 0.025% Triton X-100, 0.5 M TRIS buffer, and stained against the CD3 antigen (1:100, Dako, Denmark) or the CD11b antigen (1:100, eBioscience, USA), respectively. Nuclei were counterstained with DAPI. The number of CD3^+^ cells and CD11b^+^ cells were quantified in a Fluoview FV10i confocal microscope and normalized related to nerve area [33]. Samples from six animals per experimental group were evaluated.

### Quantification of mRNA levels of pro-inflammatory factors in sciatic nerve

At 26 weeks of age, sciatic nerves were removed, and total RNA was purified using Trizol reagent (Invitrogen, USA). One microgram of total RNA was used to perform reverse transcription with MMLV reverse transcriptase (Invitrogen) and oligo-dT primers. Real-time PCR reactions were performed to amplify the pro-inflammatory cytokines TNF-α and IL-1β using a Light-Cycler 1.5 thermocycler (Roche, USA). Relative quantifications were performed by the ΔΔCT method [30]. The mRNA level of each target gene was normalized against the mRNA levels of β-actin in the same sample. Samples from six animals per experimental group were evaluated.

### Wound induction and ulcer area measurement

At 26 weeks of age (2 weeks after the last administration of conditioned medium or vehicle), animals were anesthetized with sevoflurane vapors (Abbott, Japan), and a layer of skin (2.5 × 3.5 mm) in full thickness was surgically removed from the dorsal surface of both feet. Digital images were taken daily for 14 days, and the foot ulcer area (mm^2^) was quantified using ImageJ software as previously described [36]. Wound size for each day was defined as the area that was not re-epithelized and was normalized by the initial wound area. Six animals per experimental group were evaluated.

### Evaluation of wound re-epithelialization, angiogenesis, and cell proliferation

Two weeks after wounding, animals were euthanized by a ketamine/xylazine overdose. The wound area was excised, fixed in 4% paraformaldehyde, and then bisected through the center of the lesion to obtain the largest diameter of the wound. For determination of epidermal area and collagen deposition, 5-μm sections were stained with hematoxylin and eosin and Masson’s trichrome, respectively. Epidermal area and collagen deposition were quantified using ImageJ software as previously described [37]. For blood vessel quantification, 5-μm sections were permeabilized with 1 mg/ml digitonin and incubated with BS1-Lectin-Alexa-647 (1:100) overnight. Nuclei were counterstained with DAPI. For VE-cadherin-based vasculature analysis, 5-μm sections were blocked for 1 h in 5% FBS, 0.025% Triton X-100, 0.5 M TRIS buffer, and stained using the VE-cadherin antibody (1:100, sc-6458, Santa Cruz Biotechnology). Nuclei were counterstained with DAPI. The dermal area positive for BS1-Lectin or VE-cadherin was quantified from Z-stack images (Fluoview FV10i confocal microscope) using ImageJ software and expressed as a percent of total dermal area.

For assessment of cell proliferation inside the wound, 5-μm sections were blocked for 1 h with 5% FBS, 0.025% Triton X-100, 0.5 M TRIS buffer, and stained against the nuclear antigen Ki67 (1:400, ab15580 Abcam, UK), and nuclei were counterstained with DAPI. The number of KI67^+^ cells was quantified in a Fluoview FV10i confocal microscope and expressed as percentage of proliferating cells in the wound. Samples from six animals per experimental group were evaluated for each analysis.

### Quantification of mRNA levels of angiogenic and matrix-related factor in the wound

One week after wounding, animals were euthanized by a ketamine/xylazine overdose. Wound area was excised, and total RNA was purified using Trizol. One microgram of total RNA was used to perform reverse transcription with MMLV reverse transcriptase and oligo-dT primers. Real-time PCR reactions were performed to amplify the angiogenic factors Angiopoietin-1, PDGF, IGF-1, and VEGF-α and the matrix-related factors collagen 1 using a Light-Cycler 1.5 thermocycler as reported above. Samples from six animals per experimental group were used.

### Proteomic analysis of AD-MSC-derived conditioned media

The protein composition of conditioned media derived from DFX-preconditioned and non-preconditioned AD-MSCs obtained from four donors were analyzed as follows. Proteins were denatured and digested by trypsin as previously described [38]. Peptides were analyzed by nanoLC-MS/MS on a Q-Exactive “classic” mass spectrometer (Thermo Fisher Scientific, USA) in data-dependent mode. Three technical replicates were acquired for each biological replicate under consideration, in order to improve the accuracy of protein quantification. Data were processed in the MaxQuant/Perseus software suite (v.1.6.2) for protein identification and label-free quantification [39]. Differentially abundant proteins were assigned by performing two-sided Student’s *t* tests with S0 = 0.2 as the correction factor and permutation-based estimate of FDR (FDR < 0.05). Functional enrichment analysis of differentially abundant proteins was performed using Reactome [40], Gene Ontology (Molecular Function and Biological Process) [41], KEGG [42], and Wiki Pathways [43] databases through enrichR web server [44]. A functional category was considered enriched if presented an adjusted *p* value smaller than 0.05. The interaction network analysis of selected proteins was performed using Genemania web server [45], considering interactions related to “co-expression,” “co-localization,” “physical interactions,” “pathway,” “predicted,” and “genetic interactions” as defined by the tool.

### Statistical analysis

Quantitative data were presented as mean ± S.E.M. Comparisons between groups were performed using one-way ANOVA with Tukey post-test. Grouped, repeated measures were analyzed with two-way ANOVA with Bonferroni post-test. *p* values < 0.05 were considered statistically significant.

## Results

### AD-MSC-conditioned medium administration improves thermal and mechanical sensitivity and restores intraepidermal nerve fiber density

**Fig. 1:**
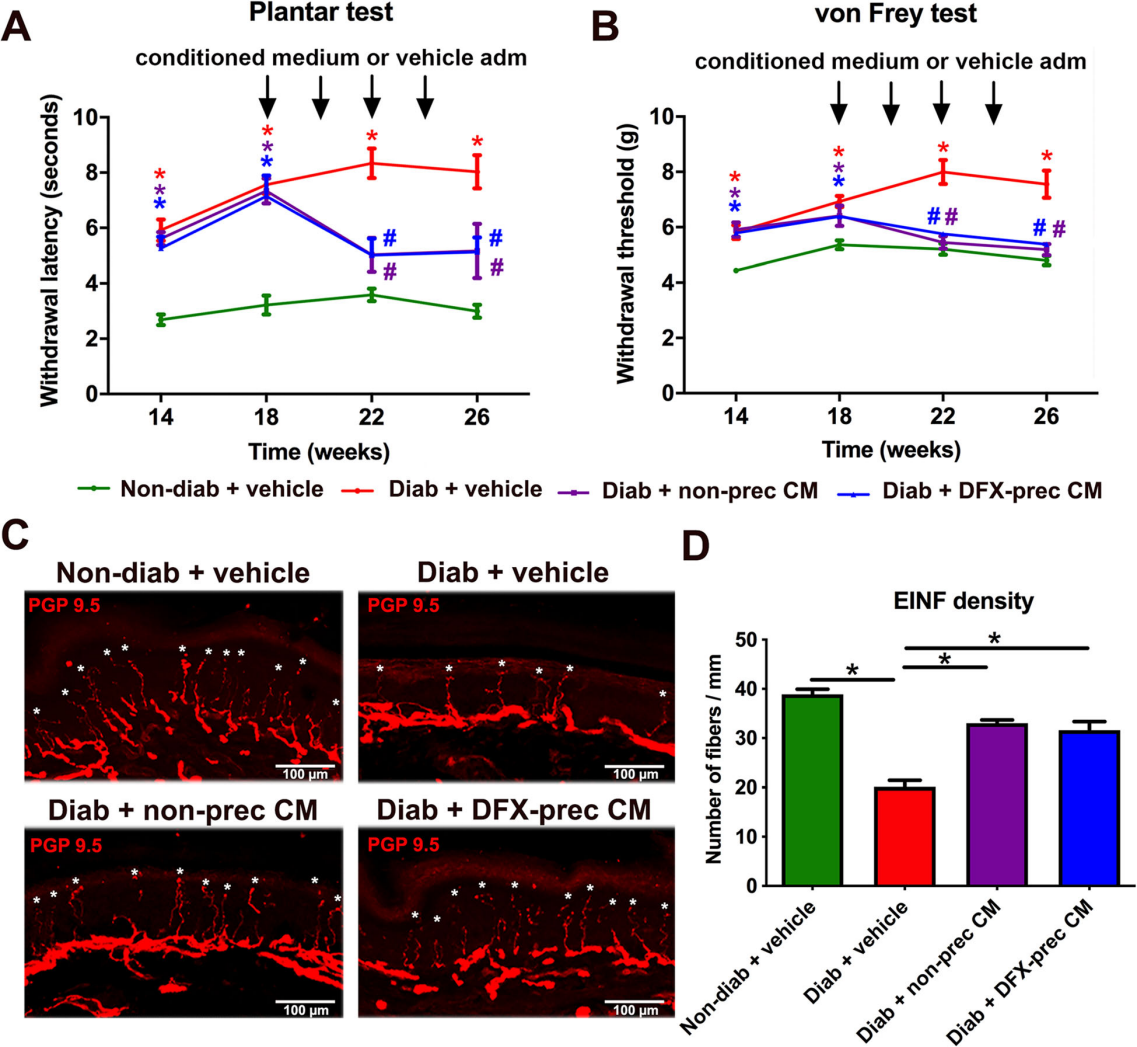
AD-MSC-conditioned medium administration improves thermal and mechanical sensitivity and restores intraepidermal nerve fiber density in diabetic mice. **a**, **b** Hargreaves plantar test (**a**) and Von Frey test (**b**) showing the withdrawal latency to thermal stimulus and withdrawal threshold to mechanical stimulus, respectively, in non-diabetic and diabetic mice treated with vehicle, conditioned medium derived from non-preconditioned AD-MSCs, or conditioned medium derived from DFX-preconditioned AD-MSCs. **c** Representative confocal images of intraepidermal nerve fibers from plantar skin samples obtained from 26-week-old non-diabetic and diabetic mice treated with vehicle, conditioned medium derived from non-preconditioned AD-MSCs, or conditioned medium derived from DFX-preconditioned AD-MSCs stained against the PGP 9.5 antigen. Asterisks indicate intraepidermal nerve fibers. **d** Quantification of the number of fibers crossing from dermis to epidermis per mm of linear skin. Data are presented as mean ± S.E.M. (*n* = 8, two-way ANOVA with Bonferroni post-test, *p < 0.05)

To evaluate the therapeutic potential of AD-MSC-derived conditioned medium, we administered systemically 50 μl of conditioned medium derived from 1 × 10^6^ DFX-preconditioned AD-MSCs or from 1 × 10^6^ non-preconditioned AD-MSCs into diabetic *db/db* mice at 18 weeks of age, when functional alterations characteristic of DPN were already present (Fig. 1a, b) [33]. Conditioned medium or vehicle administration was repeated every 2 weeks (due to the ability of its protein components), for a total of four administrations (Supplementary Figure 1).

The effect of conditioned medium administration on sensorial parameters of DPN was determined evaluating thermal and mechanical nociceptive thresholds using the plantar test and Von Frey test, respectively. We observed that administration of conditioned medium derived from DFX-preconditioned AD-MSCs or from non-preconditioned AD-MSCs to diabetic mice significantly reduced thermal hypoalgesia (Fig. 1a) and fully recovered mechanical sensitivity (Fig. 1b) compared to vehicle-treated diabetic animals.

Intraepidermal nerve fibers (IENF) are directly associated with functional innervation of the skin, and its reduction constitutes one of the main DPN histological features in humans, since it is associated with small fiber neuropathy [46]. To evaluate IENF density, we carried out an immunodetection of the PGP 9.5 antigen in cryosections of the hind limb plantar skin at 26 weeks of age, and we quantified the number of fibers crossing from the dermis to the epidermis per millimeter of linear skin. As expected, we observed that vehicle-treated diabetic animals had a significant reduction of IENF density compared to non-diabetic mice; however, the conditioned medium administration partially reversed IENF loss (Fig. 1c, d). Conditioned medium administration had no effect on blood glucose levels or body weight gain (Supplementary Figure 2A and B), suggesting that sensory improvement is a direct effect of conditioned medium administration.

Another physiological feature of DPN is the reduction of nerve conduction velocity in peripheral nerves [47]. To study the electrophysiological changes in peripheral nerves, we measured the compound motor action potential (CMAP) by stimulating the sciatic nerves of diabetic and healthy mice. Electrophysiological recordings derived from vehicle-treated diabetic mice showed decreased CMAP amplitude and increased mean peak latency (Supplementary Figure 3A-B) compared with non-diabetic mice. Conditioned medium administration induced a slightly increase in CMAP amplitude; however, we did not observe significant differences in mean peak latency (Supplementary Figure 3A-B).

### AD-MSC-conditioned medium administration recovers sciatic nerve ultrastructure

**Fig. 2:**
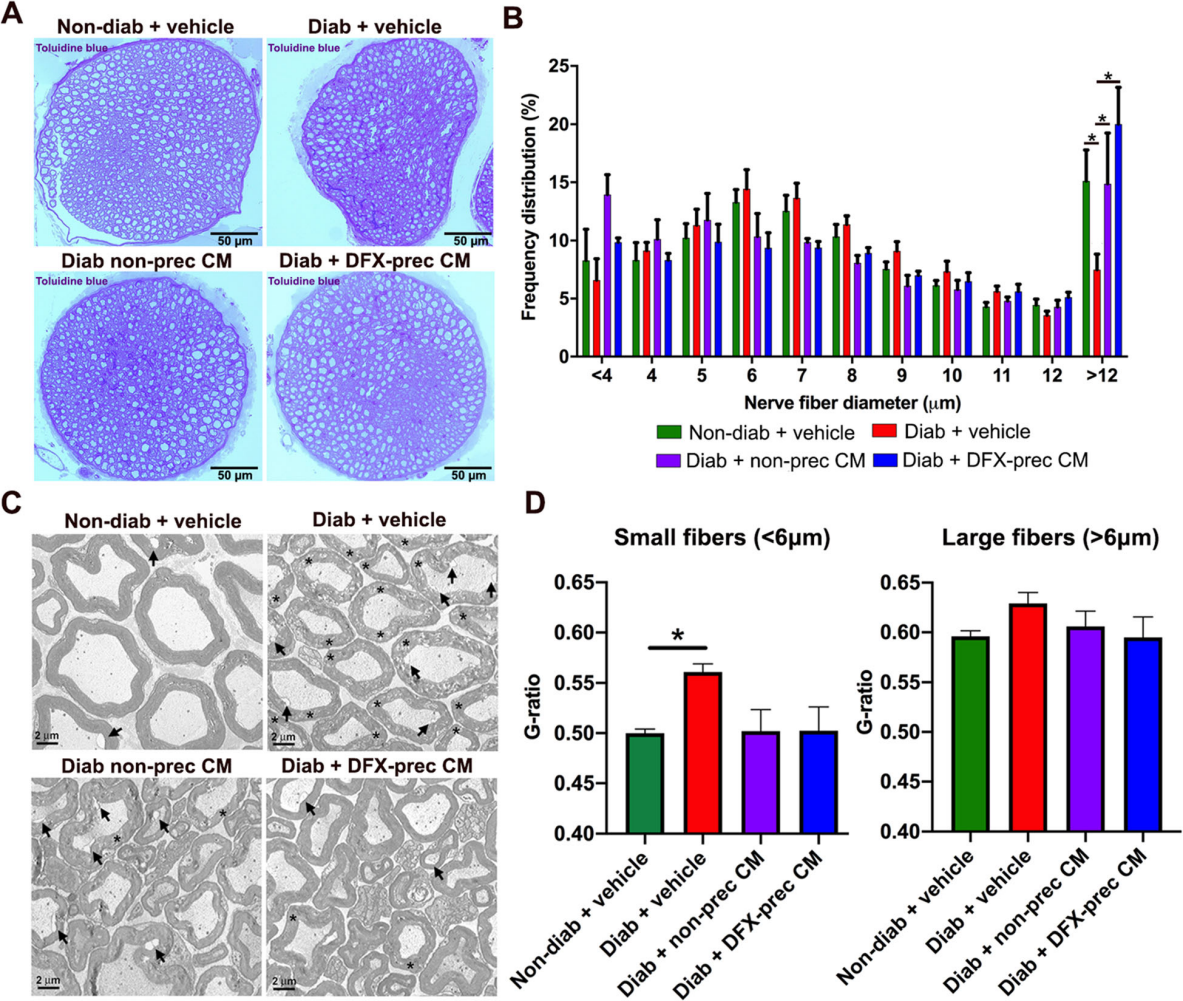
AD-MSC-conditioned medium administration recovers sciatic nerve ultrastructure in diabetic mice. **a** Representative bright-field images of semithin sections of sciatic nerves obtained from 26-week-old non-diabetic and diabetic mice treated with vehicle, conditioned medium derived from non-preconditioned AD-MSCs, or conditioned medium derived from DFX-preconditioned AD-MSCs. Myelinated fibers were stained with toluidine blue. **b** Frequency distribution histogram of myelinated fibers. **c** Representative electron microscopy images of ultrathin sections of sciatic nerves. Asterisks indicate fibers with disrupted myelin sheath, and arrows indicate the separation of shrunken axon from myelin sheath. **d** G-ratio quantification for small caliber (< 6 μm diameter) and large caliber (> 6 μm diameter) myelinated fibers of sciatic nerve. Qualitative data are presented as mean ± S.E.M. (*n* = 6, one way ANOVA with Tukey post-test, *p < 0.05)

Previous reports have indicated that both in humans and in animal models, DPN alters the morphology of nerve fibers [47]. To explore these morphological alterations, we obtained semithin sections of sciatic nerve and evaluated the size distribution of nerve fibers. As previously reported, we detected a significant decrease in the frequency of large fibers (> 12 μm diameter) in the sciatic nerve of vehicle-treated diabetic mice compared to non-diabetic mice (Fig. 2a, b). However, the administration of conditioned medium derived from DFX-preconditioned AD-MSCs or non-preconditioned AD-MSCs completely reversed the degeneration of large nerve fibers (Fig. 2a, b). At ultrastructural level, we observed that fibers from diabetic mice present some structural defects that were absent or less frequent in non-diabetic animals, including the disruption of the compacted structure of myelin sheets and the separation of shrunken axon from myelin sheath (Fig. 2c), as previously described in other animal models of DPN [21, 48–50]. These defects have been considered as early steps of neurodegeneration; meanwhile, we did not detect evident signs of axonal degeneration, such as mitochondria swelling, Schwann cell vacuolation and collapse, and myelin breakdown (Supplementary Figure 4) [51].

Though DM is not considered a demyelinating disease, it has been described that fibers form peripheral nerves suffer a progressive myelin loss in human patients and in animal models of DPN [52, 53]. To evaluate nerve fiber demyelination, we quantified the G-ratio (ratio axon diameter/nerve fiber diameter) in myelinated fibers of sciatic nerves. We observed that demyelination was more evident in small fibers, while large fibers did not seem affected. Thus, we analyzed G-ratio in small fibers (< 6 μm diameter) and large fibers (> 6 μm diameter) separately, as previously reported [53, 54]. Our results showed that G-ratio was increased specifically in small caliber fibers of diabetic animals compared to non-diabetic mice, and this demyelination was completely reversed by the administration of conditioned medium derived from preconditioned and non-preconditioned AD-MSCs (Fig. 2d). Large caliber fibers presented similar G-ratio in all experimental groups, indicating that severe myelin alterations could be observed mainly in advanced stages of the disease, as previously reported for other animal models of T2DM [34].

### AD-MSC-conditioned medium administration increases neurite regeneration in DRG neuronal cultures

**Fig. 3:**
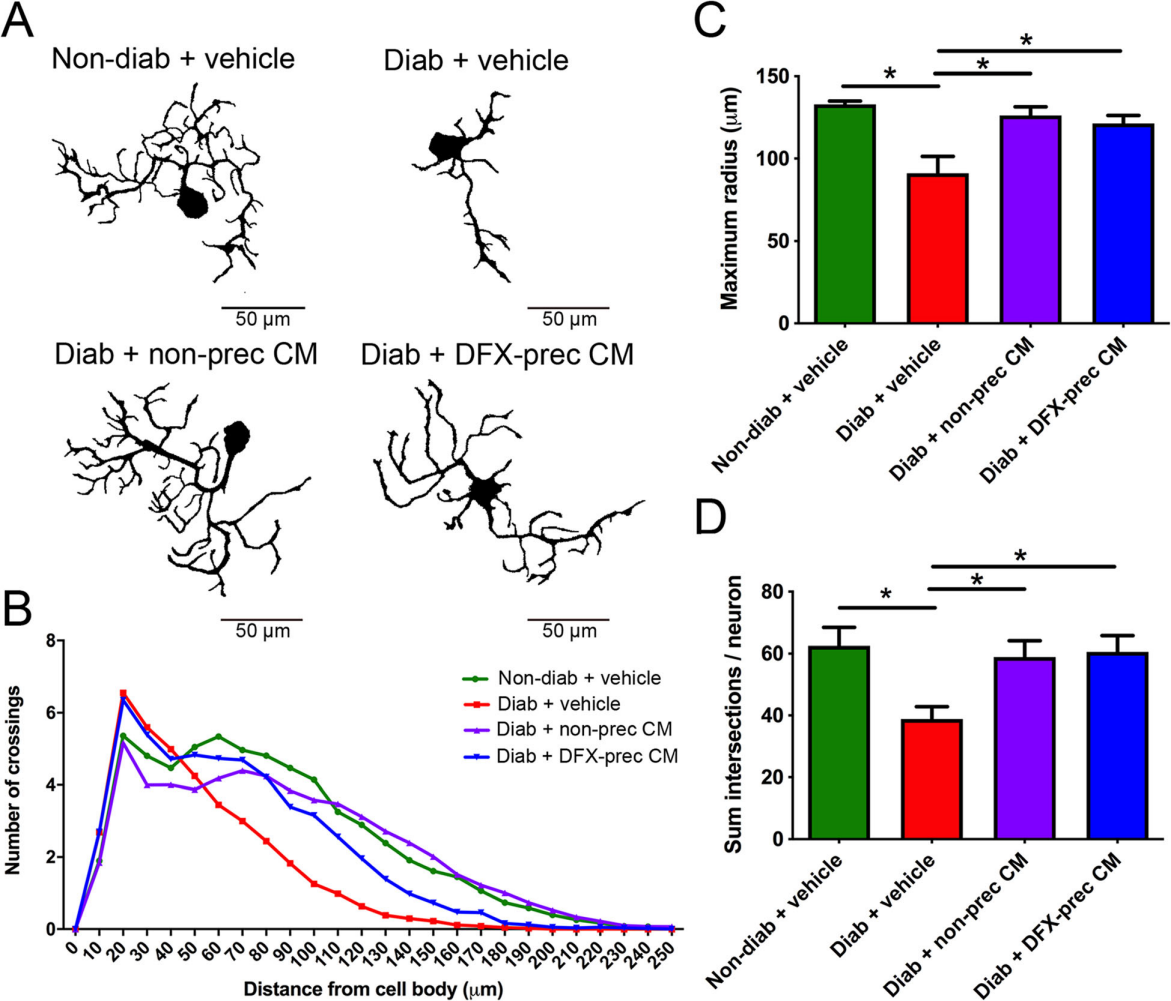
AD-MSC-conditioned medium administration increases neurite regeneration in DRG neuronal cultures derived from diabetic mice. **a** Representative binary images of DRG neurons cultured in vitro derived of non-diabetic and diabetic mice treated in vivo with vehicle, conditioned medium derived from non-preconditioned AD-MSCs, or conditioned medium derived from DFX-preconditioned AD-MSCs. **b** Sholl analysis of neurons. **c** Quantification of maximum radius and **d** number of intersections per neuron derived from Sholl analysis (*n* = 4 different cultures, 120 total neurons analyzed per condition, one-way ANOVA with Tukey post-test, *p < 0.05)

Clinical and experimental evidence indicates that axonal regeneration is highly impaired in DPN [55, 56]. To assess the contribution of in vivo AD-MSC-conditioned medium treatment to the regenerative response in sensorial neurons, we obtained DRG from conditioned medium and vehicle-treated diabetic mice and we cultured the disaggregated DRG neurons in vitro as a simplified system for studying axonal regeneration [57]. After 40 h in culture in the absence of growth factors and fetal bovine serum, DRG neurons were fixed and stained with the neuronal specific marker β3-tubulin for evaluation of neurite regeneration by Sholl analysis. We observed that DRG neurons obtained from vehicle-treated diabetic mice displayed reduced neurite length and branching compared to DRG neurons obtained from non-diabetic mice, as assessed by Sholl maximum radius and mean Sholl intersections per neuron, respectively (Fig. 3a–d). Interestingly, conditioned medium derived from DFX preconditioned AD-MSCs and from non-preconditioned AD-MSCs completely reversed the impaired regeneration phenotype associated to the diabetic condition (Fig. 3a–d).

### AD-MSC-conditioned medium administration reduces apoptosis in DRG and sciatic nerves

**Fig. 4:**
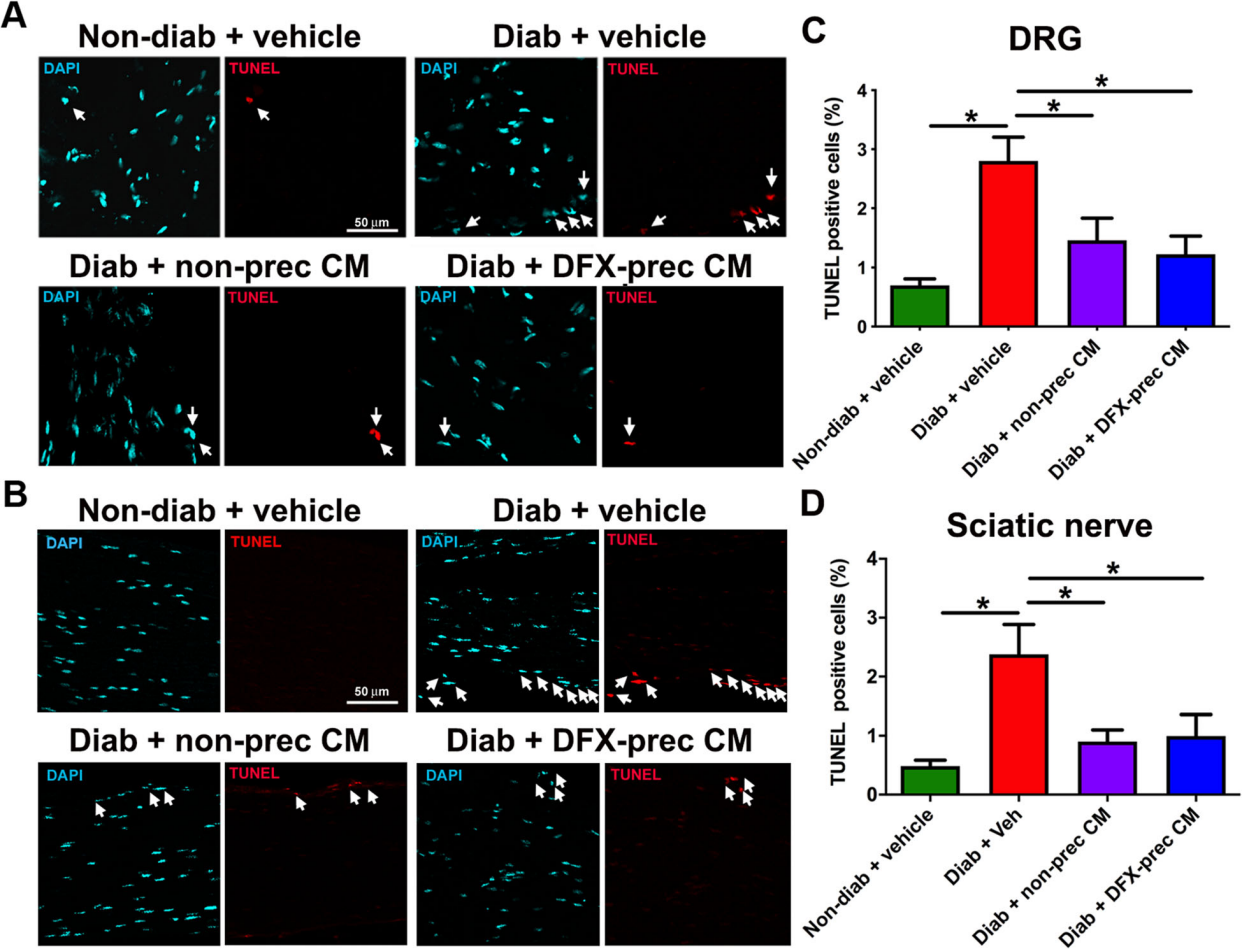
AD-MSC-conditioned medium administration reduces neuronal and Schwann cell apoptosis in DRGs and sciatic nerves of diabetic mice. **a**, **b** Representative confocal images of apoptotic cells labeled with the TUNEL staining in DRGs (**a**) and sciatic nerves (**b**), obtained from 26-week-old non-diabetic and diabetic mice treated with vehicle, conditioned medium derived from non-preconditioned AD-MSCs, or conditioned medium derived from DFX-preconditioned AD-MSCs. Nuclei were counterstained with DAPI. Arrows indicate TUNEL^+^ cells. **c**, **d** Quantification of TUNEL^+^ cells in **c** DRGs and **d** sciatic nerves. Data are presented as mean TUNEL^+^ cells ± S.E.M. (*n* = 6, one-way ANOVA with Tukey post-test, *p < 0.05)

It has been reported that due to the high mitochondrial density in neurons and Schwann cells, these cells are early affected by the oxidative microenvironment in the diabetic condition, resulting in an increased apoptotic rate [58]. To evaluate this parameter, we performed a TUNEL assay in DRGs and sciatic nerves obtained from diabetic and non-diabetic mice and quantified the number of TUNEL^+^ cells. As expected, we observed that vehicle-treated diabetic mice had a significant increase in the number of apoptotic cells at DRG and sciatic nerve levels compared to age-matched non-diabetic mice (Fig. 4a–d). Conditioned medium administration significantly reduced the number of apoptotic cells in DRG and sciatic nerve (Fig. 4a–d).

### AD-MSC-conditioned medium administration improves irrigation of peripheral nerves

**Fig. 5:**
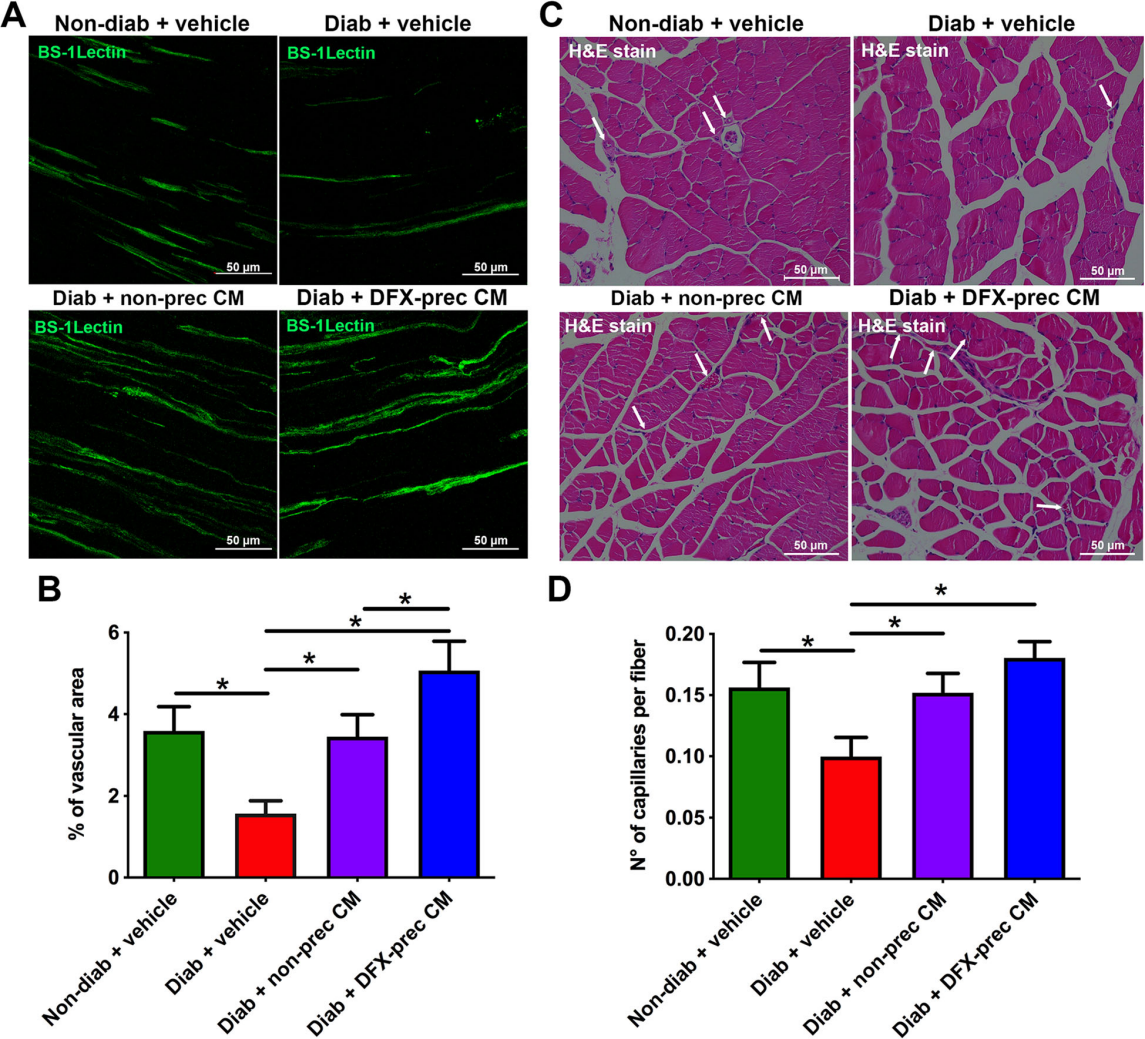
AD-MSC-conditioned medium administration improves irrigation of peripheral nerves in diabetic mice. **a** Representative confocal images of longitudinal sections of sciatic nerves obtained from 26-week-old non-diabetic and diabetic mice treated with vehicle, conditioned medium derived from non-preconditioned AD-MSCs, or conditioned medium derived from DFX-preconditioned AD-MSCs. Blood vessels were stained with BS-1Lectin. **b** Quantification of vascular area in sciatic nerves. **c** Representative bright-field images of gastrocnemius muscle sections stained with hematoxylin and eosin for blood vessel detection. Arrows indicate blood vessels. **d** Quantification of blood vessel density in gastrocnemius muscle. Data are presented as number of blood vessels per muscle fiber. Quantitative data are presented as mean ± S.E.M. (*n* = 6, one-way ANOVA with Tukey post-test, *p < 0.05)

The reduction of microvasculature of peripheral nerves is a classical hallmark of DPN in human patients [59]. Thus, we measured blood vessels in longitudinal cryosections of sciatic nerve stained with BS-1-Lectin to label endothelial cells. Additionally, the vasculature in the gastrocnemius muscle surrounding the nerve was evaluated by hematoxylin and eosin staining, and the blood vessel-to-muscle fiber ratio was determined. As expected, we observed that vehicle-treated diabetic mice had a significant reduction in blood vessel in the sciatic nerve (Fig. 5a, b) and muscle (Fig. 5c, d) compared to non-diabetic mice. However, conditioned medium administration recovered blood vessel density in both structures (Fig. 5a–d). The administration of conditioned medium derived from DFX-preconditioned AD-MSCs had a more potent effect recovering the sciatic nerve vasculature compared to non-preconditioned AD-MSC-conditioned medium-treated animals (Fig. 5a, b).

### AD-MSC-conditioned medium administration reduces sciatic nerve chronic inflammation

**Fig. 6:**
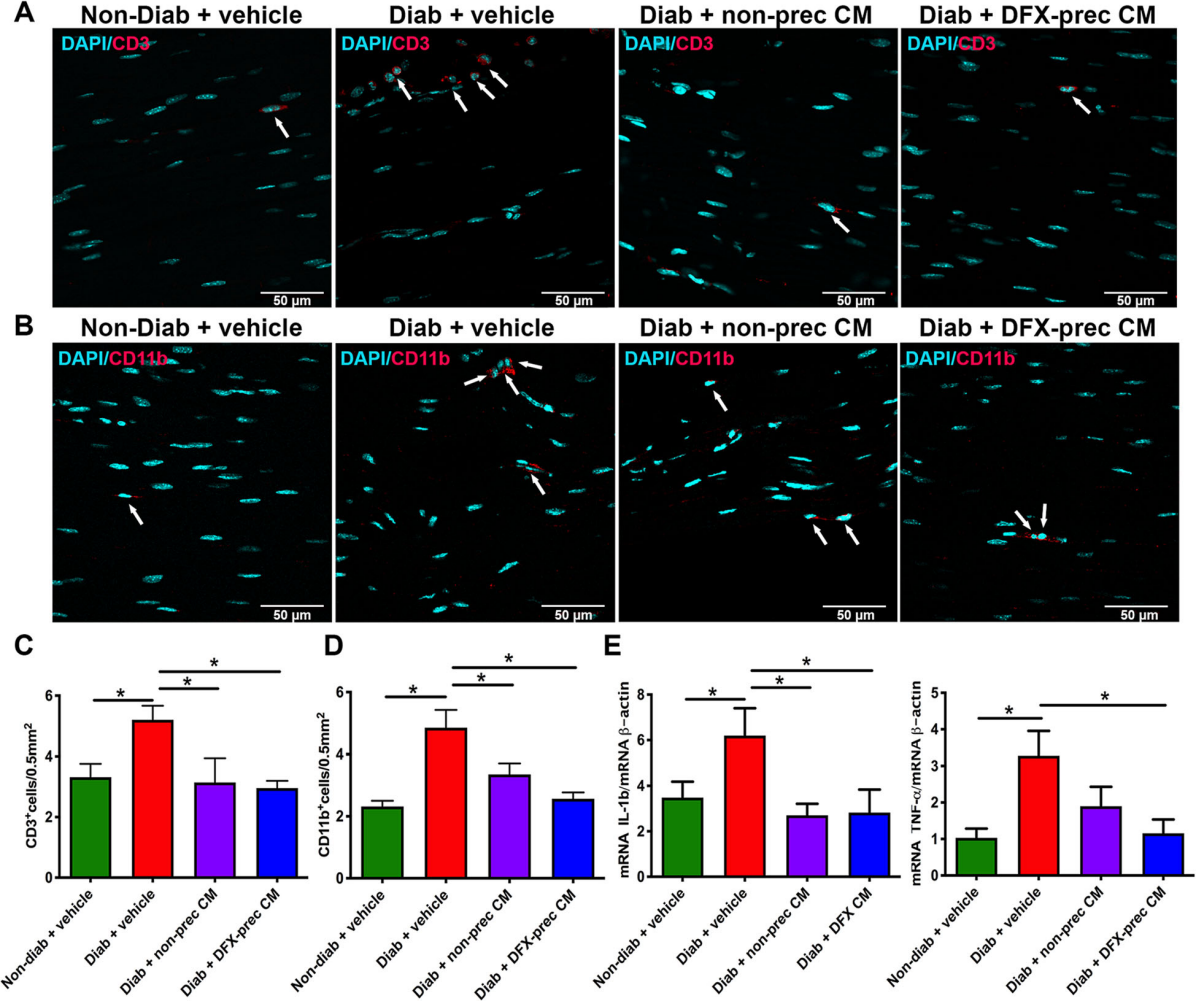
AD-MSC-conditioned medium administration reduces sciatic nerve inflammation in diabetic mice. **a**, **b** Representative confocal images of longitudinal sections of sciatic nerves obtained from 26-week-old non-diabetic and diabetic mice treated with vehicle, conditioned medium derived from non-preconditioned AD-MSCs, or conditioned medium derived from DFX-preconditioned AD-MSCs. T lymphocytes were detected with anti-CD3 antibody (**a**) while macrophages were detected with anti-CD11b antibody (**b**). Nuclei were stained with DAPI. Arrows indicates CD3^+^ cells or CD11b^+^ cells. **c** Quantification of CD3^+^ cells in sciatic nerve. Data are presented as number of CD3^+^ cells per 0.5 mm^2^ of sciatic nerve. **d** Quantification of CD11b^+^ cells in sciatic nerve. Data are presented as number of CD11b^+^ cells per 0.5 mm^2^ of sciatic nerve. **e** Quantification of mRNA levels of the pro-inflammatory cytokines IL-1β and TNF-α in the sciatic nerve. Data were normalized against the housekeeping gene β-actin. Quantitative data are presented as mean ± S.E.M. (*n* = 6, one-way ANOVA with Tukey post-test, *p < 0.05)

The generation of chronic inflammation in peripheral nerves directly contributes to nerve degeneration and chronic pain [60]. To evaluate T lymphocyte and macrophage infiltration in peripheral nerves, we performed an immunofluorescence analysis against the CD3 and CD11b antigens in sciatic nerves. A significant increase in T lymphocyte and macrophage infiltration was observed in sciatic nerves of vehicle-treated diabetic mice compared to non-diabetic mice, while the administration of conditioned medium derived from DFX-preconditioned AD-MSCs or non-preconditioned AD-MSCs restored T lymphocyte and macrophage infiltration to normal levels (Fig. 6a–d).

Following this process and in order to evaluate the possible immunomodulatory action of conditioned medium in sciatic nerves, we evaluated the production of the main pro-inflammatory cytokines by RT-qPCR. Our analysis demonstrated that vehicle-treated diabetic mice exhibited an upregulation of IL-1β and TNF-α mRNA levels relative to non-diabetic mice (Fig. 6e). However, pro-inflammatory cytokines levels were restored to normal level after conditioned medium administration (Fig. 6e).

### AD-MSC-conditioned medium administration accelerates wound healing

**Fig. 7:**
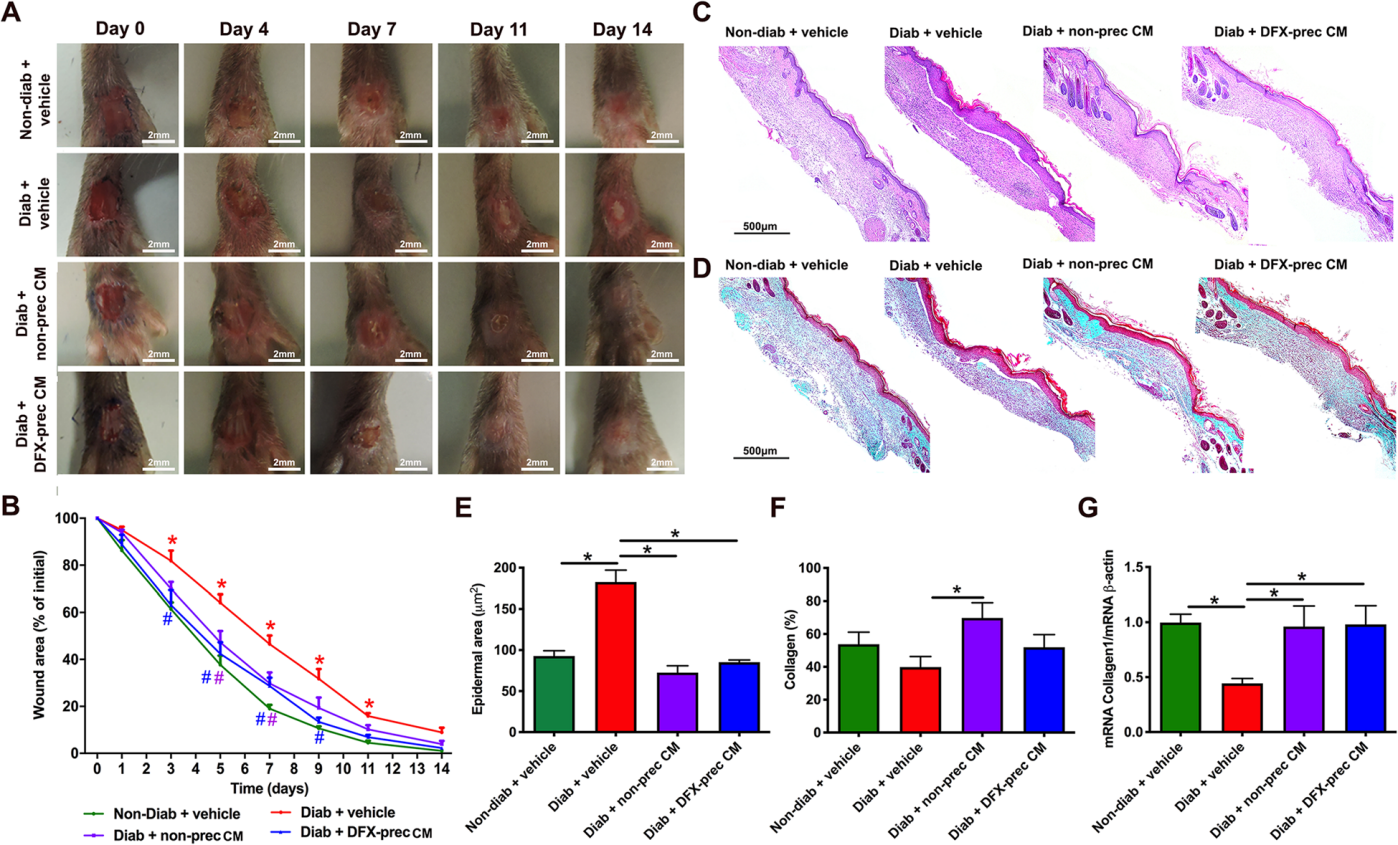
AD-MSC-conditioned medium administration accelerates wound healing in diabetic mice. **a** Representative digital photographs of the dorsal surface of the feet of 26-week-old non-diabetic and diabetic mice treated with vehicle, conditioned medium derived from non-preconditioned AD-MSCs, or conditioned medium derived from DFX-preconditioned AD-MSCs at different days after wound induction. **b** Quantification of kinetics of wound closure. Wound size for each day was defined as the area that was not re-epithelized and was normalized by the initial wound area. **c** Representative bright-field images of hematoxylin and eosin-stained histological sections of the wounds, 14 days after wound induction, for evaluation of epidermal area. **d** Representative bright-field images of Masson’s trichrome stained histological sections of the wounds, 14 days after wound induction for evaluation of collagen deposition. **e** Quantification of epidermal area in the wound bed. **f** Quantification of collagen deposition in the wound bed. **g** Quantification of mRNA levels of type I collagen in the wound bed 7 days after wound induction. Data were normalized against the housekeeping gene β-actin. Quantitative data are presented as mean ± S.E.M. (*n* = 6, one-way ANOVA with Tukey post-test, *p < 0.05)

The main complication associated to DPN is the recurrent formation of FU. To evaluate whether conditioned medium administration, in addition to reverse several hallmarks of DPN, is able to prevent the formation of FU, we wounded the dorsal surface of the hind limbs 2 weeks after the last conditioned medium administration and measured the wound area daily. We observed a significant delay in wound closure in vehicle-treated diabetic mice compared to non-diabetic mice (Fig. 7a, b). However, the previous treatment with conditioned medium derived from DFX-preconditioned AD-MSCs or non-preconditioned AD-MSCs significantly accelerated wound healing (Fig. 7a, b). Histological analysis of the wound bed was performed to further evaluate the healing of the foot skin ulcers. Fourteen days after wound induction, we measured the epidermal area and collagen deposition in the wound by hematoxylin and eosin staining and by Masson’s trichrome staining, respectively. Vehicle-treated diabetic mice presented a significant increase in wound epidermal area (Fig. 7c, e) and a significant reduction in collagen deposition in the wound bed (Fig. 7d, f). These results were correlated with a significant reduction in the expression level of type I collagen evaluated 7 days after wound induction (Fig. 7g), suggesting the presence of an altered re-epithelialization process in these animals. Conditioned medium administration completely normalized epidermal area and collagen deposition in diabetic mice (Fig. 7c–f) and restored the normal expression levels of type I collagen in the wounds (Fig. 7g).

### AD-MSC-conditioned medium administration improves the angiogenesis of the wounds

**Fig. 8:**
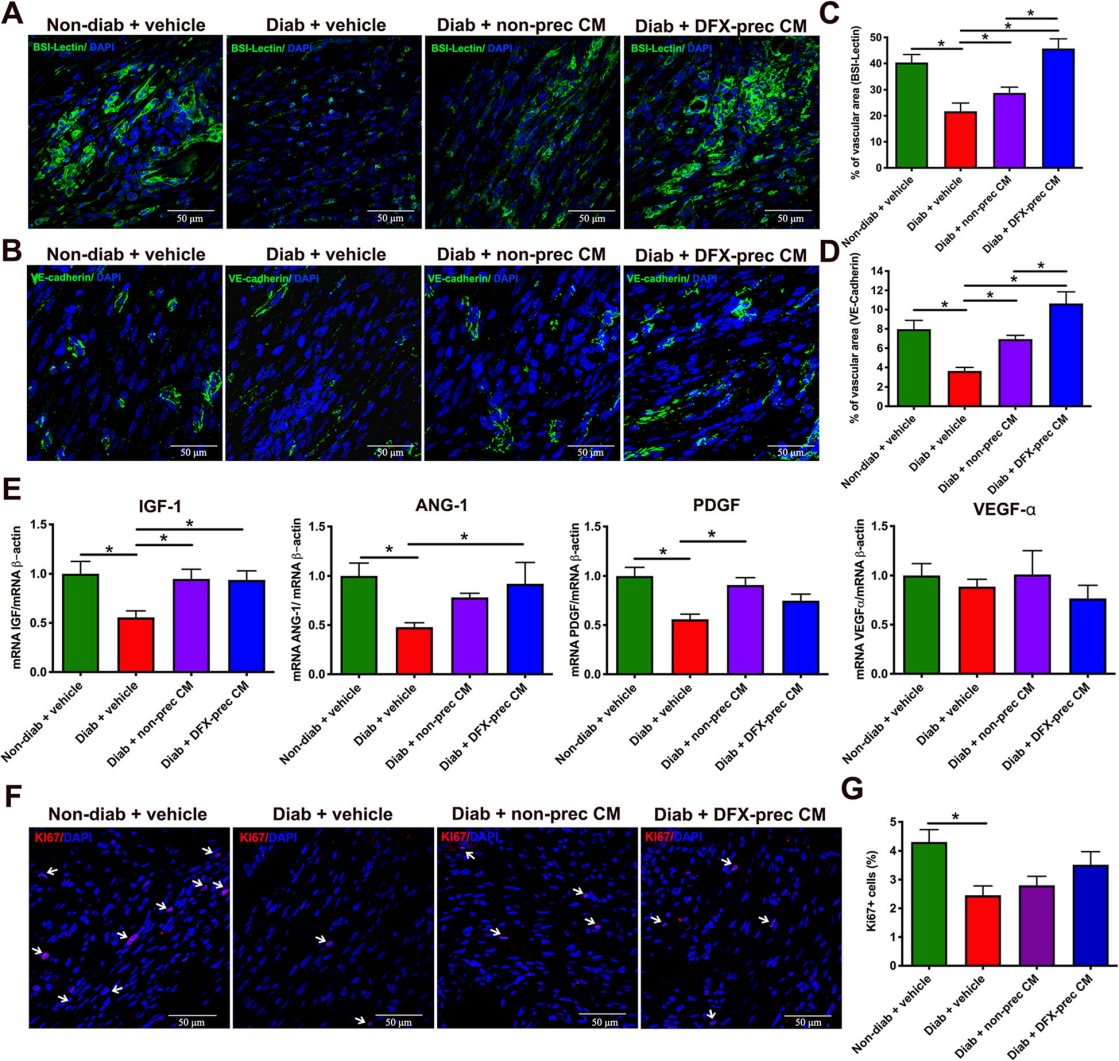
AD-MSC-conditioned medium administration improves the angiogenesis of the wounds in diabetic mice. **a**, **b** Representative confocal images of histological sections of the wound obtained from 26-week-old non-diabetic and diabetic mice treated with vehicle, conditioned medium derived from non-preconditioned AD-MSCs, or conditioned medium derived from DFX-preconditioned AD-MSCs, 14 days after wound induction. Blood vessels were stained with BS-1Lectin (**a**) or VE-cadherin (**b**) and nuclei were stained with DAPI. **c**, **d** Quantification of the vascular area in the wound 14 days after wound induction for BS1-Lectin labeling (**c**) or VE-cadherin labeling (**d**). **e** Quantification of mRNA levels of pro-angiogenic factors IGF-1, angiopotetin-1, PDGF, and VEGF-α in the wound bed, 7 days after wound induction. Data were normalized against the housekeeping gene β-actin. **f** Representative confocal images of histological sections of the wound, 14 days after wound induction. Proliferating cells in the wound were detected with anti-Ki67 antibody. Nuclei were stained with DAPI. Arrows indicates Ki67 positive cells. **g** Quantification of proliferating cells in the wound 14 days after wound induction. Data are presented as percentage of Ki67 cells per square millimeter of wound area. Quantitative data are presented as mean ± S.E.M. (*n* = 6, one-way ANOVA with Tukey post-test, *p < 0.05)

One of the main complications of diabetic wound healing is the reduced angiogenesis present in the injured skin. Thus, to better characterize the re-epithelialization process, we evaluated the vascular network in the wound by BS-1-Lectin staining, which binds to glycoproteins present in endothelial cells, and by VE-cadherin staining to specifically label endothelial cells, 14 days after wound induction. As expected, vehicle-treated diabetic mice presented a significant reduction of the wound vascular area measured by both markers compared to non-diabetic mice (Fig. 8a–d), which was correlated with a significant reduction in the expression level of several potent angiogenic factors evaluated by RT-qPCR 7 days after wound induction (Fig. 8e). In contrast, animals treated with conditioned medium derived from DFX-preconditioned AD-MSCs or non-preconditioned AD-MSCs had a significant improvement in the vascular area of the wound (Fig. 8a–d), and the expression levels of several angiogenic factors were similar to age-matched healthy mice (Fig. 8e). Once again, the administration of conditioned medium derived from DFX-preconditioned AD-MSCs had a more potent effect in recovering the skin vasculature compared to non-preconditioned AD-MSC-conditioned medium-treated mice (Fig. 8a–d).

Given that cellular proliferation in the wound tissue may contribute to ulcer healing, we next evaluated whether conditioned medium administration promoted cellular proliferation by analyzing Ki67 immunostaining. As expected, we observed that, 14 days after wound induction, vehicle-treated diabetic animals had a significant reduction in Ki-67^+^ cells in the wound bed compared to non-diabetic mice (Fig. 8f, g). Conditioned medium administration partially reversed the reduction in cell proliferation in the wound (Fig. 8f, g).

### Proteomic analysis of conditioned medium derived from non-preconditioned AD-MSCs and from DFX-preconditioned AD-MSCs

**Fig. 9:**
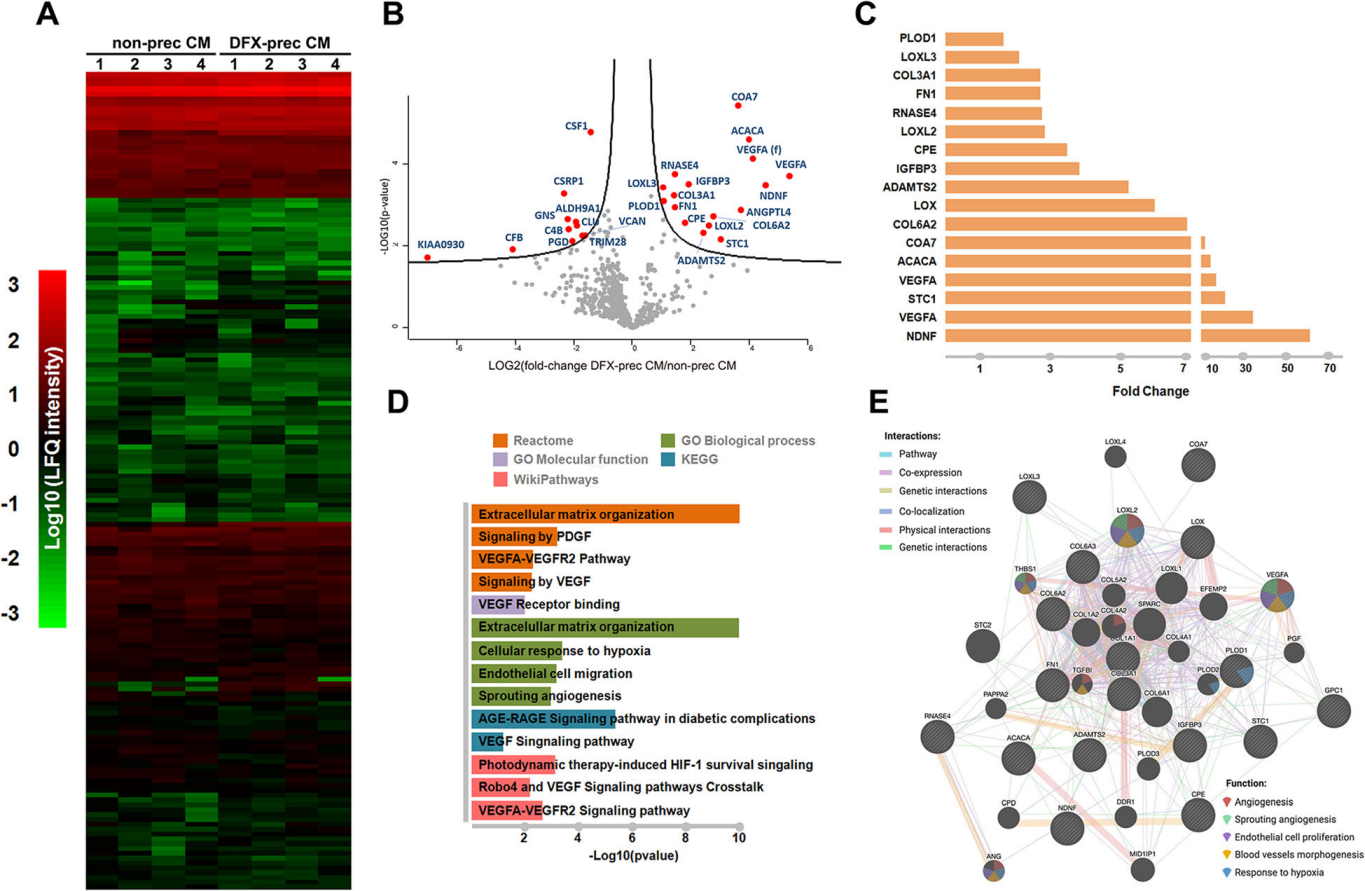
Mass spectrometry, functional enrichment, and network analysis of conditioned medium derived from non-preconditioned AD-MSCs and from DFX-preconditioned AD-MSCs. **a** Heatmap representation comparing the LFQ intensity (log10) of all 569 proteins recovered from conditioned medium derived from non-preconditioned AD-MSCs and from DFX-preconditioned AD-MSCs. **b** Volcano plot showing differentially expressed proteins between conditioned media derived from both conditions. The (f) associated to VEGF-α protein indicates a 111 amino acids (H0YBI8, UniProt) fragment different to the full length VEGF-α. **c** Histogram representing the set of 17 significantly overexpressed proteins in the conditioned medium derived from DFX-preconditioned AD-MSCs compared to the conditioned medium derived from non-preconditioned AD-MSCs. **d** Functional enrichment analysis (*p* value < 0.05) of the differentially expressed proteins according to Reactome, Gene Ontology (Molecular Function and Biological Process), KEGG, and Wiki Pathways databases. Enriched functional categories were selected based on their association with the angiogenesis process. **e** Network analysis of the selected overexpressed proteins according to different interaction categories and protein functions based on Genemania web server. The proteomic analysis was carried out using 4 different biological samples per condition

Finally, we performed a nLC-MS/MS analysis to identify differentially secreted proteins between non-preconditioned AD-MSCs and DFX-preconditioned AD-MSCs. Four conditioned media of each experimental condition were analyzed, and a total of 569 proteins were identified in both conditions. The majority of these proteins did not present differences when comparing DFX-preconditioned and non-preconditioned AD-MSCs conditions (Fig. 9a, b), indicating that both conditioned media are similar in most of its protein components. However, slight differences were attributed to 17 proteins that were found overexpressed and 11 proteins that were downregulated in the DFX-preconditioned AD-MSCs condition compared to the non-preconditioned condition (Fig. 9b, c). In order to recover additional information related to the functional roles that these differentially abundant proteins could be involved in, we performed an overrepresentation enrichment analysis using different databases related to function, pathways, and biological processes. The overrepresentation analysis revealed a significant enrichment in proteins associated with angiogenesis, response to hypoxia, extracellular matrix organization, and endothelial cell migration (Fig. 9d), which are highly related to the improved vasculogenic effect of the conditioned medium derived from DFX-preconditioned AD-MSCs in sciatic nerves and skin wounds. Additionally, network analysis revealed a high connectivity within some hub proteins presenting functional associations with “angiogenesis,” “sprouting angiogenesis,” “endothelial cell proliferation,” “blood vessels morphogenesis,” and “response to hypoxia” (Fig. 9e). However, more research is needed to identify which are the more relevant molecules behind the observed therapeutic effects.

## Discussion

Diabetic polyneuropathy is a clinical complication of DM that evolves from functional peripheral nerve impairments in the early phases of the disease toward structural changes at advanced stages [61]. Using one of the most relevant animal models of T2DM [31–33], the present study demonstrated that the systemic administration of conditioned medium derived from DFX-preconditioned AD-MSCs or from non-preconditioned AD-MSCs effectively blocked the main functional and structural features associated with DPN by modulating different pathways, once the main alterations associated to DPN were already established. Furthermore, DPN reversion induced by conditioned medium administration enhanced the wound healing process, which could be useful as a preventive treatment for FU formation in diabetic patients.

The pathogenesis of DPN is multifactorial, involving both metabolic and vascular components. However, at molecular level, the increase in ROS level in mitochondria due to exacerbated glucose flux has been proposed as the central player in DPN onset and progression [7]. Increased ROS levels lead to a severe deficiency of angiogenic and neuroprotective factors and to an increase in pro-inflammatory factors, which finally result in the generation of nerve structural damage at several levels [7]. Thus, a therapeutic approach simultaneously targeting the angiogenic, neurodegenerative, inflammatory, and oxidative process may have great value in DPN treatment.

In recent years, MSCs have emerged as a tool with great clinical potential in the treatment of multifactorial diseases. These cells can be isolated from a wide range of tissue [62]. Nevertheless, adipose tissue has been positioned as the preferred source for MSC isolation due to is accessibility, since it is considered a waste material of cosmetic liposuctions and has a higher yield of MSCs compared to other sources [63].

MSC therapy has shown promising results in the treatment of DPN at pre-clinical level [18, 20, 21]. However, limited cell survival after transplantation, due to the noxious microenvironment, the non-specific organ entrapment, the possible differentiation of MSCs into ectopic structures, the elimination of the cells by the host immune system, and possible tumorigenicity hinders its transfer from bench to bedside [64].

The broad spectrum of molecules secreted by MSCs is known as conditioned medium. In humans, the MSC-conditioned medium consists of hundreds of biologically active molecules including anti-inflammatory cytokines, trophic factors, and antioxidant molecules [65]. In fact, the remarkable therapeutic potential of MSC-derived conditioned medium has been consistently demonstrated in several experimental models, including nervous system disorders and a number of painful physical conditions [66–69]. Thus, it has been proposed that conditioned medium administration could mimic the therapeutic effects observed after the administration of MSCs, while at the same time reducing the therapeutic complexity and the potential unwanted effects associated with the administration of living cells [66–69]. However, a major issue associated with the use of MSC-conditioned medium is that in several cases, the concentration of relevant molecules is too low for therapeutic applications.

It has been reported that, due to the high plasticity of MSCs, it is possible to modify the MSC-conditioned medium baseline composition by subjecting these cells to an in vitro preconditioning stimulus, thus producing a biological product with a defined combination and ratio of biomolecules specific for a determined pathology [28, 70]. In this context, we recently reported that the preconditioning of AD-MSCs with the hypoxic mimetic agent deferoxamine increased the level of the hypoxia-inducible factor HIF1α, which activates several intracellular pathways that finally result in a significant increase in the secretion of several potent angiogenic, neuroprotective, anti-inflammatory, and antioxidant factors [30], which could enable the concerted effect necessary for neurovascular recovery in DPN.

Our data indicate that systemic administrations of conditioned medium derived from DFX-preconditioned MSCs and form non-preconditioned MSCs were able to significantly relieve thermal and mechanical hypoalgesia in diabetic mice, restoring the normal sensitivity threshold compared to that of untreated diabetic mice. To correlate the improvement in behavior sensory neuropathy with a reduction in sensory small nerve fiber damage, we analyzed intraepidermal nerve fiber density at the footpads. Cutaneous sensory neurons include unmyelinated C-fiber and myelinated Aδ-fibers and Aβ-fibers [71], while thermal and mechanical sensitivity are determined mainly by C-type small fibers and myelinated Aδ-fibers [71]; all these fibers could be labeled using the PGP 9.5 antigen. We observed that the administration of both conditioned medium were able to ameliorate the loss of PGP-9.5^+^ nerve fibers. Interestingly, in human patients, a strong correlation between sensory loss and reduced PGP 9.5^+^ intraepidermal nerve fiber density has been described, and intraepidermal nerve fiber density is negatively correlated with diabetes duration [72]. Further immunofluorescence analysis with markers for specific fibers would be important in order to assess which fibers are lost and recovered after conditioned medium administration.

As previously reported in human patients [73] and in animal models [47, 74], diabetic mice displayed a significant decrease in large diameter nerve fibers (> 12 μm diameter) in the sciatic nerve, which are characterized by a lower electrical threshold and a higher conduction velocity. Thus, the reduction in CMAP amplitude and prolonged motor latencies observed in untreated diabetic mice could be related to the drop out of large myelinated axons and therefore a reduction in the number of motor units responsible for the smaller size of the CMAP. The administration of conditioned medium derived from DFX-preconditioned AD-MSCs and from non-preconditioned AD-MSCs completely prevented the loss of large fibers, prevented common ultra-structural alterations, and restored the normal G-ratio in small diameter fibers of the sciatic nerve. However, it had a partial effect in restoring the normal electrophysiological properties, suggesting that additional factors may also contribute to the nerve conduction alterations in diabetic mice. It has been reported that another mechanism involved in saltatory propagation in myelinated nerve fibers is the resting membrane potential that becomes hyperpolarized as the result of an inhibition of internodal inward rectification induced by hyperglycemia [75]. Thus, even though axonal diameter improves in diabetic mice treated with conditioned medium administrations, this mechanism may explain the lack of conduction velocity improvement in these animals.

Neurons and Schwann cells are especially sensitive to the damage induced by ROS, which leads to cell apoptosis [58]. By secreting a broad range of neuroprotective factors, Schwann cells play a central role in neuron survival but also in axonal regeneration of peripheral nerves after injury [76]. Therefore, the early death of these cells in DPN induces a significant reduction of the local levels of neuroprotective factors, generating a vicious cycle that enhances nerve fiber degeneration and prevents their regeneration [77]. Using DRG neurons in culture as a simplified system for studying axonal regeneration [57], we observed that conditioned medium administration completely reversed the impaired regeneration phenotype associated with the diabetic condition compared to neurons obtained from vehicle-treated diabetic mice. Furthermore, the administration of conditioned medium derived from DFX-preconditioned AD-MSCs or from non-preconditioned AD-MSCs significantly reduced the apoptosis of neurons and Schwann cells in vivo. These effects could be related to the presence of several neurotrophic and antiapoptotic factors including BDNF, NGF, and NT3 in the conditioned medium [30].

Increased ROS levels induce alterations in endothelial cells, generating loss of the vasodilatation capacity of epineural blood vessels and reduction of angiogenic factors production. This phenomenon leads to a reduction in the microcapillaries that irrigate nerve fibers, with the induction of local ischemia and neuronal dysfunction [59]. Thus, impairment of peripheral blood flow is one of the major factors triggering DPN [59]. In our model, DPN induced a significant reduction in blood vessel density in sciatic nerves and a marked reduction in the capillary-to-muscle fiber ratio compared to that of healthy animals. However, conditioned medium administration fully restored the normal microvasculature, which could be related to the presence of potent angiogenic factors in the conditioned medium, since the administration of conditioned medium derived from DFX-preconditioned AD-MSCs, which is significantly enriched in angiogenic factors [30], induced a better angiogenic response compared to non-preconditioned AD-MSC-conditioned medium-treated animals.

Accumulating evidence also suggests the involvement of inflammatory process in the pathogenesis of DPN [60, 78]. Increased oxidative stress leads to activation of the poly ADP-ribose polymerase (PARP) pathway, which is responsible for regulating pro-inflammatory cytokine expression [60]. This creates a chronic inflammatory state that exerts direct toxic effects on neurons and Schwann cells, accelerating the atrophy of nerve fibers and contributing to the chronic pain characteristic of DPN patients [60]. We observed that vehicle-treated diabetic mice had a significant increase in T lymphocyte and macrophage infiltration that was correlated with a significant increase in the expression levels of the pro-inflammatory cytokines TNF-α and IL1β, which have been associated with chronic pain [79]. However, after the administration of conditioned medium derived from DFX-preconditioned AD-MSCs or from non-preconditioned AD-MSCs, neuroinflammation was significantly blunted, with T lymphocyte and macrophage infiltration and pro-inflammatory cytokine production restored to normal levels. These results are in accordance with previous studies in diabetic animals indicating that MSCs administration alters the inflammatory response by shifting the cytokine profile to an anti-inflammatory phenotype [80, 81].

Successful wound healing is a dynamic and complex process including a series of coordinated events such as inflammation, cell migration, proliferation, differentiation, angiogenesis, and re-epithelialization [82]. However, several of these processes are severely affected in diabetic patients [83]. Thus, chronic lower extremity wounds have become the most common and severe complication of DM.

Our data indicates that the previous administration of conditioned medium derived from DFX-preconditioned AD-MSCs and from non-preconditioned AD-MSCs significantly accelerates the ulcer healing on the foot of diabetic mice. This result was accompanied with the restoration of the normal epithelial thickness after wound closure and the improvement in collagen expression level and collagen deposition in the wound bed of conditioned medium-treated diabetic mice compared to vehicle-treated diabetic mice.

The paramount importance of blood supply in the healing of wounds has long been appreciated, since it is necessary to sustain and enhance newly formed granulation tissue, which is essential to wound healing [83]. Diabetic animals presented a significant reduction in capillary density in the wound bed and a significant reduction in the expression levels of angiogenic factors. However, these alterations were fully reversed in diabetic mice treated with conditioned medium derived from DFX-preconditioned AD-MSCs.

Keratinocyte and fibroblast proliferation and migration into the wound is also an important factor in the re-epithelialization process [84]. We observed that the diabetic microenvironment significantly reduced cell proliferation in the wound. However, this effect was partially reversed in conditioned medium-treated diabetic mice. Additional research is needed in order to know the contribution of conditioned medium administration in other process affected during wound healing that could be contributing to the improvement in wound closure observed in conditioned medium treated diabetic mice.

There are some reports showing that the administration of MSCs or MSCs-derived conditioned medium directly onto the wounds of diabetic animals improved wound closure by promoting skin re-epithelialization and improving the angiogenic process [85–87]. However, since FU constantly occur in diabetic patients, this complex procedure needs to be repeated whenever the patient has a new ulcer. Here, we propose a different therapeutic approach in which the systemic administration of conditioned medium at initial stages of DPN could reverse functional and structural alterations of peripheral nerves, avoiding the generation of FU, but also accelerating the closure of the wound in case it occurs.

In our study, no significant changes in blood glucose levels was observed after conditioned medium administration, suggesting that the conditioned medium-mediated relief of functional and structural alterations characteristic of DPN and the improvement in wound healing are independent of glycemia.

Recently, we reported that conditioned medium obtained from DFX-preconditioned AD-MSCs showed greater neuroprotective effects compared with the conditioned medium obtained from non-preconditioned AD-MSCs, when evaluated in an in vitro model of DPN [30]. Here, our data showed that when conditioned media were evaluated in an animal model of DPN, both were equally potent in reversing the main functional and structural alterations characteristic of DPN.

The main therapeutic differences between both conditioned media were associated to the improvement of the irrigation of the sciatic nerve and the wound bed in the injured skin, in which the administration of conditioned medium derived from DFX-preconditioned AD-MSCs exhibited a superior effect compared to the administration of conditioned medium derived from non-preconditioned cells, suggesting that preconditioned AD-MSCs may support the angiogenic process better than non-preconditioned cells. Compared with different preconditioning stimulus, DFX has the advantage that this drug has been used in humans for three decades with no toxic effects to treat iron overload diseases [88], and it has been previously demonstrated that due to its small size, it could be completely removed from the final product in the concentration steps [30].

The content and relevance of the factors present in the AD-MSC-conditioned medium is still under study. Our proteomic analysis identified 569 factors, which are mostly shared by both conditioned media (Fig. 9a, b). This fact could explain the similarities in the therapeutic effects achieved by both conditioned media. Among these 569 factors, 17 were significantly overrepresented in the conditioned medium derived from DFX-preconditioned AD-MSCs, including potent angiogenic factors such as VEGF-α and NDNF, which could explain the superiority of this conditioned medium to potentiate the angiogenesis process compared to conditioned medium derived from non-preconditioned AD-MSCs. Other proteins highly represented, such as stanniocalcin-1 and VEGF-α (f) (a 111 amino acid fragment, H0YBI8 in UniProt), also constitutes proteins with an established role in angiogenic sprouting and putative VEGF receptor ligand, respectively [89, 90].

Selective neutralization of individual factors as well as combinations of factors will allow the definition of the essential components required to induce the therapeutic effects in DPN treatment. If specific paracrine MSC-derived factors can be identified, then protein-based therapy might be used.

## Conclusions

This research indicates that the cocktail of bioactive mediators present in the conditioned medium of AD-MSCs could convert a neurodestructive/pro-inflammatory microenvironment to a neuroprotective/anti-inflammatory one. Thus, using a conditioned medium of MSCs may be an excellent and novel therapeutic method to reverse the initial stages of DPN, avoiding the generation of FU and reducing the risk of lower limb amputation.

## Supplementary information

**Additional file 1 : Supplementary Figure 1**: Experimental Design. At 18 weeks of age, diabetic (*db/db*) mice were randomly divided in three groups. The first group was treated with intravenous administration of 50 μl of conditioned medium derived from 1 × 10^6^ DFX-preconditioned AD-MSCs. The second group was treated with intravenous administration of 50 μl of conditioned medium derived from 1 × 10^6^ non-preconditioned AD-MSCs, while the third group received intravenous administration of 50 μl of vehicle. Conditioned medium or vehicle administrations were repeated every two weeks for a total of four administrations. An additional group of normal non-diabetic (*db/+*) mice treated with vehicle was used as a healthy control. At 18, 22 and 26 weeks of age, functional parameters characteristic of DPN including hypoalgesia to thermal and mechanical stimulation and motor nerve conduction velocity were evaluated. At 26 weeks of age, some animals of each group were euthanized and several structural markers characteristic of DPN including intraepidermal nerve fiber density, ultrastructural analysis of nerve fibers, apoptosis of neurons and Schwann cells, blood flow to nerve fibers and infiltration of T lymphocytes and macrophages were evaluated. At 26 weeks of age the remaining animals were anesthetized and a wound was made in the dorsal surface of both feet. Wound closure was evaluated daily while determination of mRNA levels of angiogenic, and matrix-related factors and determination of wound re-epithelialization, angiogenesis, and cell proliferation was evaluated 7 days and 14 days respectively after wound induction.

**Additional file 2 : Supplementary Figure 2**: Plasma glucose level and body weight is not altered by conditioned medium administration. (A) Blood glucose levels were evaluated every two weeks using a glucometer. (B) Body weight was evaluated every two weeks. Data are presented as mean ± SEM (*n* = 20 per experimental group, two-way ANOVA with Bonferroni post-test).

**Additional file 3 : Supplementary Figure 3**: Electrophysiological impairment of sciatic nerve is partially rescued by conditioned medium administration. (A) Quantification of CMAP amplitude in non-diabetic and diabetic mice treated with vehicle, conditioned medium derived from non-preconditioned AD-MSCs, or conditioned medium derived from DFX-preconditioned AD-MSCs measured at 22 and 26 weeks of age. (B) Quantification of CMAP latency-to-peak of the same animals of Fig. A measured at 22 and 26 weeks of age. Data are presented as mean ± S.E.M. (*n* ≥ 8, one-way ANOVA with Tukey post-test, * *p* < 0.05).

**Additional file 4 : Supplementary Figure 4**: Mitochondrial area and density in sciatic nerves is not affected by diabetic condition. (A) Representative electron microscopy images of ultrathin sections of sciatic nerves, showing mitochondria (arrows) structure and distribution in myelinated and unmyelinated fibers. (B) Quantification of mitochondria density and area in myelinated and unmyelinated fibers. Data are presented as mean ± S.E.M. (*n* = 6, one way ANOVA with Tukey post-test).

## Data Availability

All data generated or analyzed during this study are included in this published article and its supplementary information file.

## Abbreviations

α-MEM: Minimal essential medium alpha
AD-MSCs: Adipose tissue-derived mesenchymal stem cells
BDNF: Brain-derived neurotrophic factor
CD3: Cluster of differentiation 3
CD11b: Cluster of differentiation 11b
CM: Conditioned medium
CMAP: Compound motor action potential
DAPI: 4′,6-Diamidino-2-phenylindole
DFX: Deferoxamine
DM: Diabetes mellitus
DPN: Diabetic polyneuropathy
DRG: Dorsal root ganglia
FBS: Fetal bovine serum
FU: Foot ulcers
HIF1α: Hypoxia-inducible factor 1 alpha
IENF: Intraepidermal nerve fibers
IGF-1: Insulin-like growth factor*-*1
IL-1β: Interleukin 1 beta
LC-MS: Liquid chromatography–mass spectrometry
MSCs: Mesenchymal stem cells
NDNF: Neuron derived neurotrophic factor
NGF: Neural growth factor
NT3: Neurotrophin 3
PARP: Poly ADP-ribose polymerase
PBS: Phosphate-buffered saline
PDGF: Platelet-derived growth factor
PGP-9.5: Protein gene product 9.5
ROS: Reactive oxygen species
TNF-α: Tumor necrosis factor alpha
TUNEL: Terminal deoxynucleotidyl transferase dUTP nick end labeling
VEGF-α: Vascular endothelial growth factor alpha
