# Supplementary figures and images for: Human adipose-derived mesenchymal stem cell-conditioned medium ameliorates polyneuropathy and foot ulceration in diabetic BKS db/db mice
Source: Stem Cell Res Ther. 2020 May 1;11:168. doi: 10.1186/s13287-020-01680-0 (PMC7195803; doi:10.1186/s13287-020-01680-0)

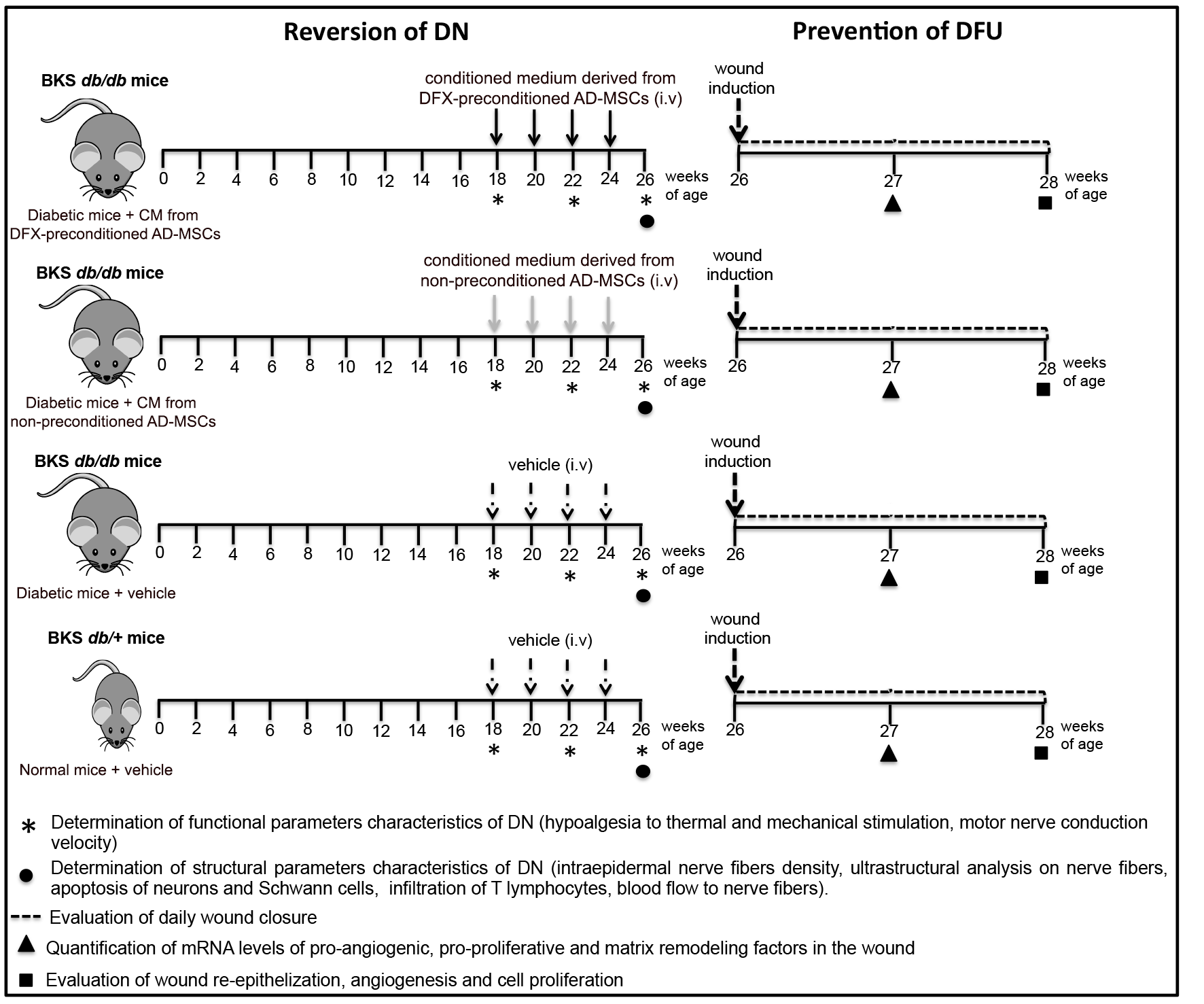

Supplement: Supplementary file 1 — Additional file 1 : Supplementary Figure 1: Experimental Design. At 18 weeks of age, diabetic (db/db) mice were randomly divided in three groups. The first group was treated with intravenous administration of 50 μl of conditioned medium derived from 1 × 10^6^ DFX-preconditioned AD-MSCs. The second group was treated with intravenous administration of 50 μl of conditioned medium derived from 1 × 10^6^ non-preconditioned AD-MSCs, while the third group received intravenous administration of 50 μl of vehicle. Conditioned medium or vehicle administrations were repeated every two weeks for a total of four administrations. An additional group of normal non-diabetic (db/+) mice treated with vehicle was used as a healthy control. At 18, 22 and 26 weeks of age, functional parameters characteristic of DPN including hypoalgesia to thermal and mechanical stimulation and motor nerve conduction velocity were evaluated. At 26 weeks of age, some animals of each group were euthanized and several structural markers characteristic of DPN including intraepidermal nerve fiber density, ultrastructural analysis of nerve fibers, apoptosis of neurons and Schwann cells, blood flow to nerve fibers and infiltration of T lymphocytes and macrophages were evaluated. At 26 weeks of age the remaining animals were anesthetized and a wound was made in the dorsal surface of both feet. Wound closure was evaluated daily while determination of mRNA levels of angiogenic, and matrix-related factors and determination of wound re-epithelialization, angiogenesis, and cell proliferation was evaluated 7 days and 14 days respectively after wound induction. [file 13287_2020_1680_MOESM1_ESM.tif]

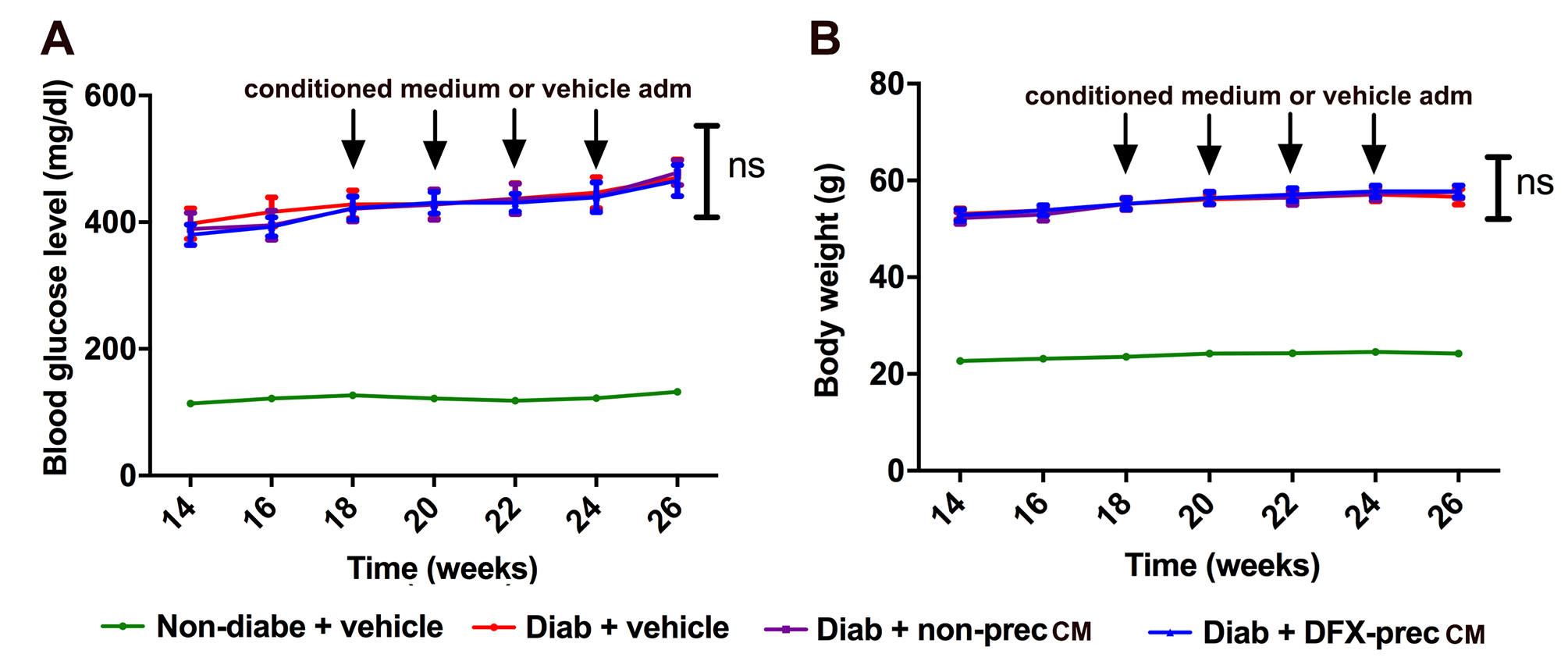

Supplement: Supplementary file 2 — Additional file 2 : Supplementary Figure 2: Plasma glucose level and body weight is not altered by conditioned medium administration. (A) Blood glucose levels were evaluated every two weeks using a glucometer. (B) Body weight was evaluated every two weeks. Data are presented as mean ± SEM (n = 20 per experimental group, two-way ANOVA with Bonferroni post-test). [file 13287_2020_1680_MOESM2_ESM.tif]

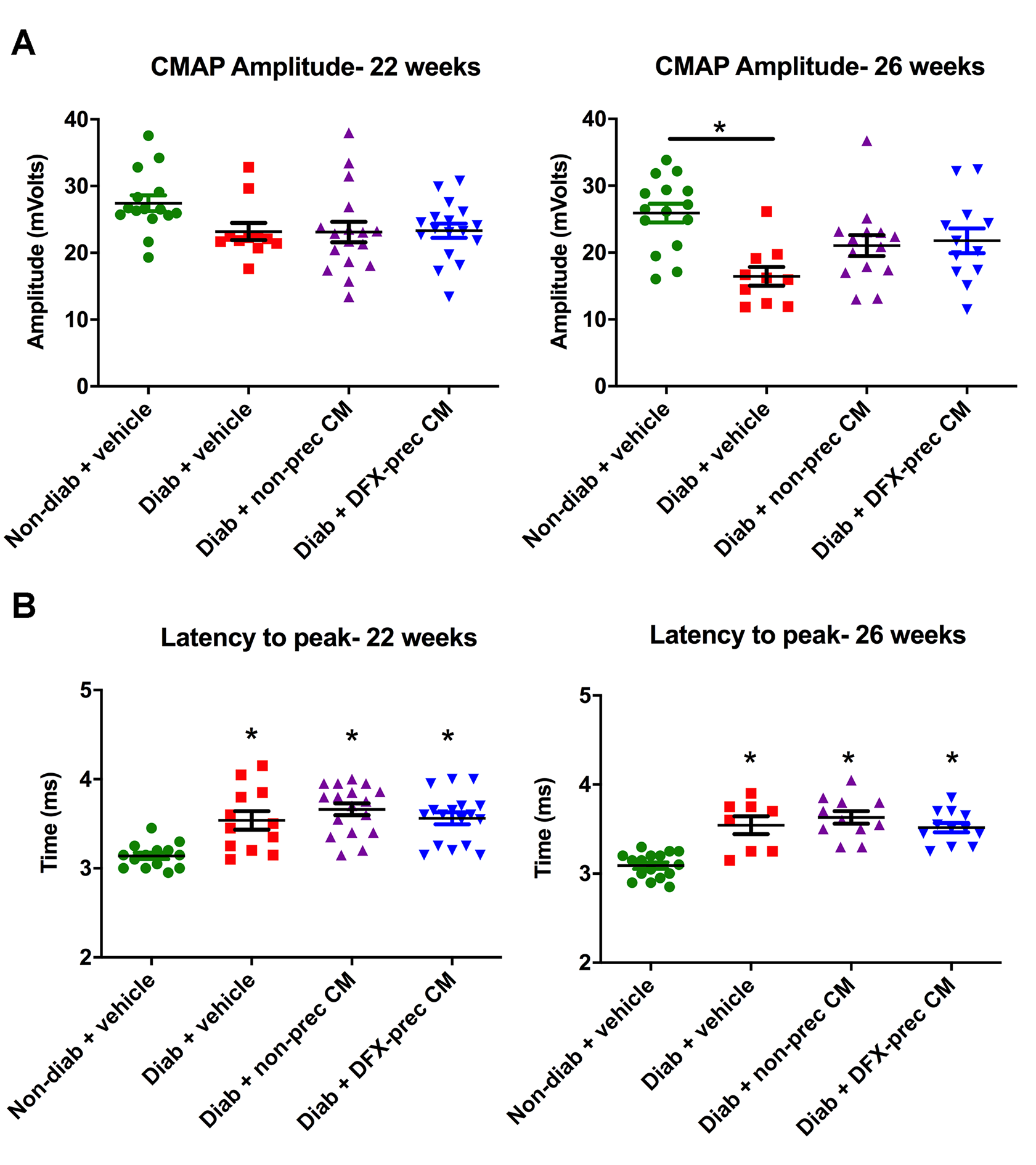

Supplement: Supplementary file 3 — Additional file 3 : Supplementary Figure 3: Electrophysiological impairment of sciatic nerve is partially rescued by conditioned medium administration. (A) Quantification of CMAP amplitude in non-diabetic and diabetic mice treated with vehicle, conditioned medium derived from non-preconditioned AD-MSCs, or conditioned medium derived from DFX-preconditioned AD-MSCs measured at 22 and 26 weeks of age. (B) Quantification of CMAP latency-to-peak of the same animals of Fig. A measured at 22 and 26 weeks of age. Data are presented as mean ± S.E.M. (n ≥ 8, one-way ANOVA with Tukey post-test, * p < 0.05). [file 13287_2020_1680_MOESM3_ESM.tif]

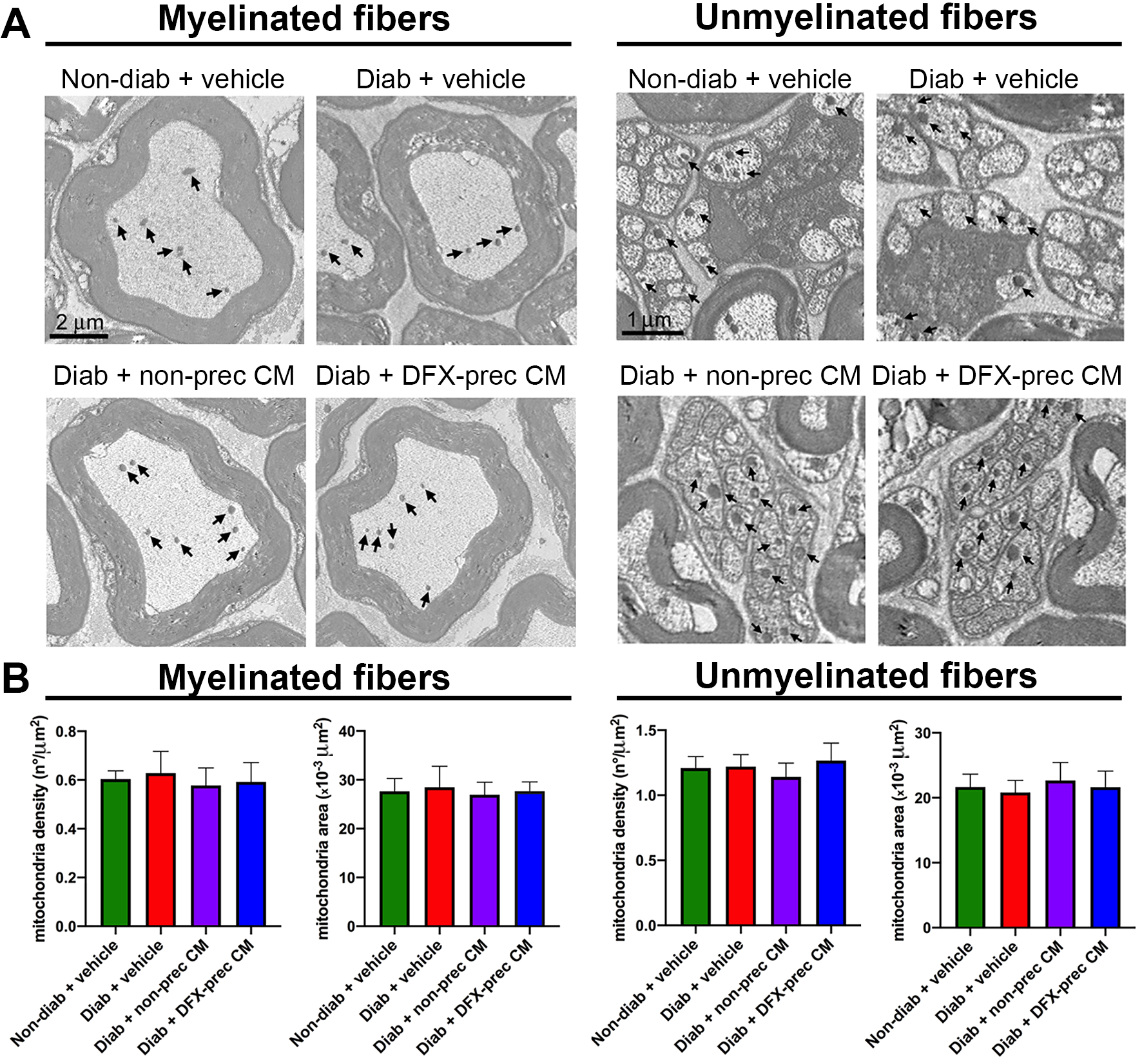

Supplement: Supplementary file 4 — Additional file 4 : Supplementary Figure 4: Mitochondrial area and density in sciatic nerves is not affected by diabetic condition. (A) Representative electron microscopy images of ultrathin sections of sciatic nerves, showing mitochondria (arrows) structure and distribution in myelinated and unmyelinated fibers. (B) Quantification of mitochondria density and area in myelinated and unmyelinated fibers. Data are presented as mean ± S.E.M. (n = 6, one way ANOVA with Tukey post-test). [file 13287_2020_1680_MOESM4_ESM.tif]
